# Computational promoter analysis of mouse, rat and human antimicrobial peptide-coding genes

**DOI:** 10.1186/1471-2105-7-S5-S8

**Published:** 2006-12-18

**Authors:** Manisha Brahmachary, Christian Schönbach, Liang Yang, Enli Huang, Sin Lam Tan, Rajesh Chowdhary, SPT Krishnan, Chin-Yo Lin, David A Hume, Chikatoshi Kai, Jun Kawai, Piero Carninci, Yoshihide Hayashizaki, Vladimir B Bajic

**Affiliations:** 1 Knowledge Extraction Laboratory, Institute for Infocomm Research, 21 Heng Mui Keng Terrace, Singapore 119613, Singapore; 2 Department of Biochemistry, Faculty of Medicine, National University of Singapore, 8 Medical Drive, Singapore 117597, Singapore; 3 Immunoinformatics Research Team, Advanced Genome Information Technology Group, RIKEN Genomic Sciences Center (GSC), RIKEN Yokohama Institute, 1-7-22 Suehiro-cho, Tsurumi-ku, Yokohama, Kanagawa, 230-0045, Japan; 4 Division of Genomics and Genetics, School of Biological Sciences, Nanyang Technological University, Singapore 637551, Singapore; 5 Department of Obstetrics and Gynecology, National University Hospital, National University of Singapore, 5 Lower Kent Ridge Road, Singapore 119074, Singapore; 6 University of the Western Cape, South African National Bioinformatics Institute (SANBI), Private Bag X17, Bellville 7535, South Africa; 7 Brigham Young University, Department of Microbiology and Molecular Biology, 753 WIDB, Provo, UT 84602, USA; 8 ARC Special Research Centre for Functional and Applied Genomics, Institute for Molecular Bioscience, The University of Queensland, Brisbane, Queensland 4072, Australia; 9 Genome Exploration Research Group (Genome Network Project Core Group), RIKEN Genomic Sciences Center (GSC), RIKEN Yokohama Institute, 1-7-22 Suehiro-cho, Tsurumi-ku, Yokohama, Kanagawa, 230-0045, Japan; 10 Genome Science Laboratory, Discovery Research Institute, RIKEN Wako Institute, 2-1 Hirosawa, Wako, Saitama, 351-0198, Japan

## Abstract

**Background:**

Mammalian antimicrobial peptides (AMPs) are effectors of the innate immune response. A multitude of signals coming from pathways of mammalian pathogen/pattern recognition receptors and other proteins affect the expression of AMP-coding genes (AMPcgs). For many AMPcgs the promoter elements and transcription factors that control their tissue cell-specific expression have yet to be fully identified and characterized.

**Results:**

Based upon the RIKEN full-length cDNA and public sequence data derived from human, mouse and rat, we identified 178 candidate AMP transcripts derived from 61 genes belonging to 29 AMP families. However, only for 31 mouse genes belonging to 22 AMP families we were able to determine true orthologous relationships with 30 human and 15 rat sequences. We screened the promoter regions of AMPcgs in the three species for motifs by an *ab initio *motif finding method and analyzed the derived promoter characteristics. Promoter models were developed for alpha-defensins, penk and zap AMP families. The results suggest a core set of transcription factors (TFs) that regulate the transcription of AMPcg families in mouse, rat and human. The three most frequent core TFs groups include liver-, nervous system-specific and nuclear hormone receptors (NHRs). Out of 440 motifs analyzed, we found that three represent potentially novel TF-binding motifs enriched in promoters of AMPcgs, while the other four motifs appear to be species-specific.

**Conclusion:**

Our large-scale computational analysis of promoters of 22 families of AMPcgs across three mammalian species suggests that their key transcriptional regulators are likely to be TFs of the liver-, nervous system-specific and NHR groups. The computationally inferred promoter elements and potential TF binding motifs provide a rich resource for targeted experimental validation of TF binding and signaling studies that aim at the regulation of mouse, rat or human AMPcgs.

## Background

Antimicrobial peptides (AMPs) comprise an important component of the innate immune system in protecting the host from microorganisms. Mammals produce many different antimicrobial peptides that are active against a broad spectrum of pathogens, including gram-positive and gram-negative bacteria, protozoans, fungi and some viruses [1]. The AMPs may either exhibit their antimicrobial activity directly as gene encoded products or after processing from longer precursor proteins by proteolytic cleavage. Many AMPs are also involved in functions that are not directly associated with the innate immune response. Under normal physiological conditions hepcidin is an important regulator of iron homeostasis in the liver and macrophages [2,3], but it can also acts as microbicidal and fungicidal AMP [4]. Another AMP, the neutrophil granule derived peptide cap37, which binds to gram-negative bacterial endotoxins, can also act as signaling molecule causing the up-regulation of protein kinase C activity [5].

Individual AMPs may have distinct functions in different locations, for example at mucosal surfaces or in phagocytes, and must be differentially regulated depending on the presence or absence of a pathogen challenge. AMPs may also need to be expressed in a concerted manner. Although AMPs are intensely studied on protein level [6-8] data and progress on transcriptional control mechanism of AMPs is limited to a few families such as beta-defensins and cathelicidins [9,10]. Therefore, we aim in this study at the computational identification of AMP promoter elements (PEs), followed by the characterization of commonalities and differences of PEs among AMPcg families within one species and across different species. Since the study was conducted within the framework of the FANTOM3 [11,12] project, our sequence sources are RIKEN mouse full-length cDNAs (flcDNAs). These sequences were used to extract the promoter regions from mouse alpha-defensin, apoa2, beta-defensin, bpi, spag11, cathelicidin, calgranulin, dbi, slpi, granulin, hepcidin, histone2a, lactoferrin, lysozyme, mbp, melanotropin alpha, proenkaphalin, secretogranin, spyy, vasostatin, vip and zap AMPcg families and their human and rat orthologs.

## Results and Discussion

### Extraction of AMPcgs and their promoter sequences

**Figure 1:**
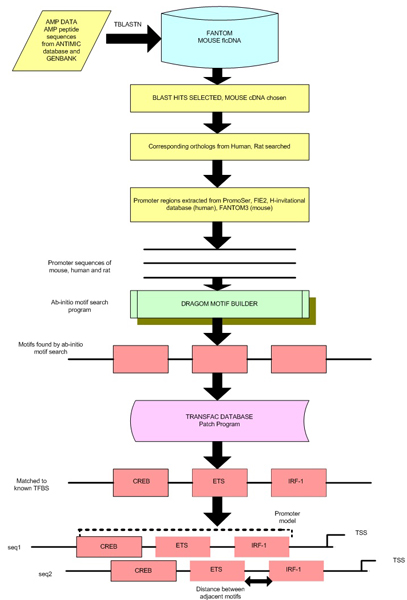
**Workflow of promoter sequence set preparation and analysis**. AMP peptide sequences were collected from ANTIMIC and Genbank databases and searched with TBLASTN against FANTOM3 cDNA sequences applying a cut-off of equal or greater than 60% identity. The promoter regions [-1000, +200 nt] of mouse AMPcg, human and rat orthologs were extracted and submitted to Dragon Motif Builder (DMB) for *ab initio *motif searching. The resulting consensus motifs were passed to TRANSFAC and compared with known TFBSs using the PATCH program.

The initial steps of this AMPcg promoter study comprise the identification of AMPcg cDNAs in the FANTOM3 data set and their orthologous human or rat sequences. AMPcg transcripts can be identified by keyword, gene ontology term, motif or sequence similarity searches or combinations thereof. Since the identification of AMPcg RIKEN mouse flcDNAs started during the FANTOM3 annotation when gene names and gene ontology were not yet stable, we extracted candidate sequences using TBLASTN [13] sequence similarity search against a set of known AMP sequences (Fig. 1) [14]. Of 183 mouse candidates with sequence identities equal or greater than 60% to known AMPs over the length of 100 residues and with E-values of 0.01 or less, five were recognized as false positives by checking their stable gene name and gene ontology annotations. In total, we identified 178 AMPcg sequences. When subtracting previously published FANTOM1 and 2 sequences we obtained 103 mouse AMPs members that were new in FANTOM3. The sequences belonged to 28 families (alpha-defensin, alpha2casein, apoa2, beta-defensin, spag11, bpi, calgranulin, cathelicidin, cathepsinG, dbi, slpi, enhancer of rudimentary homolog, granulin, hepcidin, histone2a, IFN-inducible antiviral protein Mx, lactoferrin, lysozyme, mbp, melanotropin alpha, ovotransferrin, proenkephalin 1, sap2, secretogranin, skiv2l, spyy, vasostatin, vip and zap). The majority of new mouse AMP-coding cDNAs were derived from macrophage, adipocyte and testis cDNA libraries.

The definition of true orthology across species is difficult in multigene families associated with innate immunity, wherein gene duplication is a common feature of evolution. For example, we found that S100 (calgranulin) has three human myeloid-associated family members S100A8, A9 and A12, but only two (S100A8 and A9) members in mice [15]. Similarly, we noticed that the mouse AMP casein delta (csnd), defensin-related sequence cryptidin peptide (Defcr-rs1), mast cell protease family (mcpt2, mcpt4, mcpt8), and histone2a (Hist2h2aa2), did not have the corresponding family members in human (Supplementary Table 1, Additonal File 1). On the other hand, the Rnase A family member Rnase 7 was found in human, but was absent in mouse.

We restricted our analysis to mouse FANTOM3, rat and human sequence data because our approach aimed at finding differences and similarities among mammalian orthologs of mouse. Orthologs of mouse genes in frog, fish or invertebrates are too distant for promoter analyses and often lack accurate promoter sequence data. Therefore we considered only a subset of *bona fide *orthologous mouse, human and rat promoter sequences representing only 22 out of 29 AMP families. Thirty-one promoter region sequences were derived from mouse, 30 from human orthologs and 15 from rat orthologs (Supplementary Table 2, Additional File 2). Mouse cryptidins were included in the alpha-defensin family because they represent a subfamily of alpha-defensins [16].

### *Ab initio *motif discovery in AMPcg promoter sequences

A commonly applied method for identifying motifs in promoter regions of co-regulated genes utilizes predetermined position-weight matrices for known TF binding sites (TFBSs) of TRANSFAC [17], JASPAR [18] and other databases. Another popular method for discovering motifs enriched in co-regulated genes is biclustering of genes and conditions [19]. In this study the *ab initio *motif discovery method was used because it permits the sequence context-dependent identification of both new and known TFBS motifs. Although there are several *ab initio *motif discovery programs available [20], none of them showed a distinct advantage over the others on all data types. Therefore, we compared the performances of DMB [21], an in-house developed program with two other programs, MEME [22] and Improbiser [23]. All three programs use *ab initio *motif discovery algorithms based on expectation maximization. We used the promoter sequences of the proenkephalin (penk) AMP group (4922504O09, HIX0007519.2, NM_017139), which has been studied empirically in transfection assays. Penk promoters are known to possess a TATA box and respond to cyclic AMP, glucocorticoids and protein kinase C (AP1) agonists [24,25]. Since Improbizer identified only six motifs, we first considered the top six motifs produced by each of these systems. Among the top six motifs, DMB- reported three motifs (TATA, AP-2, AP-1) that may bind TFs known to control the penk promoter [26,27]. MEME reported one motif (TATA) and Improbiser two (NF-Y, TATA) motifs. Since DMB and MEME can identify arbitrary number of motifs, we also compared the top 20 motifs generated by DMB and MEME. Seven DMB-derived motifs coincided with known TFBSs (TATA, NF-kappaB, AP-2, AP-1 NFI/CTF, NF-Y, MZF1, MIG1, MBP-1) [26,27] known to control penk promoter. MEME yielded only three known penk promoter motifs (TATA, NFI/CTF, AP-1). Considering the differences in performance and the longer computation time of MEME we used DMB throughout the entire analysis. The *ab initio *determined known and new motifs and their distribution among AMPcg families are shown in Supplementary Table 3, Additional File 3 and in Table 1, respectively. Forty-one (59%) out of 70 experimentally confirmed AMPcg family-associated TFs may bind to predicted known DMB-derived motifs (Supplementary Table 3, Additional File 3). For each AMP family, motifs were discovered that did not match any of the known TRANSFAC-contained motifs and were reported as "unknown motifs". Another set of motifs matched to known TFBS but were previously not reported to control AMPcgs. These new AMPcg-associated candidates are shown in Table 1.

### Over-represented TF binding motifs that are conserved among AMPcg families

The transcriptional regulation of AMPcg families varies from family to family because of the different tissue cell-specific expression and AMP characteristics. Thus, one would not expect that different AMPcg families show significant similarities in their promoter element organization. To test whether similarities exist and which TFs may control more than one AMPcg family we searched for shared AMPcg family motifs (see Methods). We found eight shared motif groups among 94 motif instances that present 31 mouse, 30 human and 15 rat AMP promoter sequences (Supplementary Table 4, Additional File 4). These results indicate the existence of a core TF set that may be part of the transcription activation mechanism in the examined AMPcg families of all three species.

Each of the motif families is represented by a consensus motif obtained from all motif instances in that family. The consensus motif AGGAAA is known to be recognized by the TFs PEA3, c-Ets1, E74A, PU.1, LyF-1, c-Ets-2, ISGF-3, NF-AT1, NF-AT2, NF-AT4 and DEAF-1. Consensus motifs ACAGCA and ATGGAG are specific for GR and Nkx2-1, respectively. Consensus motif CCCGCCCC corresponds to binding site for TFs Sp1/Sp3. TGGCATT is recognized by NF-1.

The four consensus motifs found in mouse, rat and human corresponded to published and experimentally confirmed AMPcg-associated TFs. The GR transcription factor motif ACAGCA was conserved among 32 genes of ten different AMPcg families in mouse, rat and human. PEA3, c-Ets1, PU.1, LyF-1, c-Ets-2, NF-AT1, NF-AT2 and NF-AT4-specific motif AGGAAA was observed in 34 genes belonging to 11 AMPcg families. Sp1 and Sp3-specifc motif CCCGCCCC appeared in 15 genes derived from six AMPcg families. NF-1 motif TGGCATT was present in 36 genes of nine AMPcg families (see Supplementary Table 4, Additional File 4). Consensus motif CCAGGG was observed in 24 genes of eight AMPcg families; ACCTGG was present in 28 genes of seven AMPcg families; TCTTTC motif occurred in 26 genes of nine AMPcg families. These findings imply the presence of common PEs that may form part of a core transcription initiation control program for AMPcg families.

Another four motifs appeared to be species or lineage-specific in the context of regulation of individual AMPcg familes, but we cannot draw general conclusion on this issue due to the limited dataset. For example, the motif AGGAAA occurred only in three rodent genes of the lysozyme family, but not present in the human. CCAGGG was absent in genes of the human Spag11 family. TGGCATT motif was absent in human genes of the Apoa2 and Spyy families. CCCGCCCC was not found in mouse genes of the Apoa2 family (see Supplementary Table 4, Additional File 4). Similar species-specific differences were reported for the promoter of mouse and human Toll-like receptor 3 and its expression pattern [28]. Since our observations were made for the region of [-1000, +200] nucleotides (nt) of the promoters we cannot exclude the possibility of AMPcg regulation by different promoter regions in mouse and human. Due to lack of sufficient data on microbial context, signaling pathways and TF binding-data on AMPs, it remains to be seen whether these disparities reflect an exposure to a different microbial environment or physiological differences. Despite the differences in functions of AMPcg families and tissue cell-specific expression, their promoters share a number of common known and new motifs (see Supplementary Table 4, Additional File 4). Among the new motifs are CCAGGG, ACCTGG and TCTTTC that did not match to any known TFBS in TRANSFAC and JASPER databases. Only in yeast the motif TCTTTC was shown to be associated in a ChIP experiment with cell cycle specific transcription factor Spf1 [29]. Whether the new motifs are cis-elements that interact with unknown or known mouse TFs remains to be established in experiments.

### TF groups that are significantly associated with AMPcg families

To determine TF groups that are significantly associated with AMPcg families, we analyzed the TF binding motifs and the distribution of the corresponding TFs across the 22 AMPcg families. The AMPcg-associated TFs were grouped into ten tissue-specific categories (adipocyte-related, immune cell-specific, liver cell-specific, lung cell-specific, muscle cell-specific, nervous system-related, pancreatic beta cell-specific, pituitary gland-specific, eye-specific, and bone/teeth) and two general categories of cell-cycle specific TFs and nuclear hormone receptors (NHRs). Table 2 and Supplementary Table 5A, Additional File 5 show the distribution of motifs identified by DMB across all AMP families.

For each of the AMPcg family only the top two-ranked TF categories were taken into account. The ranking was based on the proportion of motifs that potentially bind TFs of a specific TF group in any AMPcg family. We considered cases when TF-binding motifs associated with a particular TF group occurred in 25%, 30%, 35% or 40% of all motifs observed in the AMPcg family. Three TF categories (liver-specific, neuron system specific, NHR) appeared to be either the first or second ranked in three out of four considered cases (25%, 30%, 35% or 40%), and these TF categories also represent the top ranked ones, overall. The results are summarized in Supplementary Table 5B, Additional File 5.

When considering the rank position of a particular TF group in individual AMPcg families, six TF categories emerge as dominant categories (Supplement Table 5B, Additional File 5). These are, in order, liver-specific, neuron system-specific, adipocyte-specific, NHR, immune cell-specific and lung-specific TFs. This ranking is obtained by using the average rank position of the TF group in each of the AMPcg family. The ranking of the TFs suggests that the functions of AMPs extend far beyond antimicrobial actions as mediators in energy metabolism and neuroendocrine regulations. The finding is reminiscent of the multi-functionality of cytokines (i.e. IL6, TNF-alpha, MIF etc.) in adipocytes, liver and immune cells during metabolic challenges and stress [30,31].

Several groups reported on the role of dihydroxyvitamin D3 [32] and glucocorticoids [33,34] in the transcription regulation of AMPcgs. Since these studies focused only on a few NHR members and few AMPcg families, the appearance of NHRs in the top-ranked TF groups among many AMPcg families was unexpected. NHR family proteins function as dimeric molecules in the nucleus to regulate the transcription of target genes in a ligand-responsive manner [35,36]. If we require that at least 35% (seven out of 20) of the identified motifs for each of the AMPcg families can bind TFs from a particular group, NHR and neuron system specific TFs appear in eleven (alpha-defensin, lactoferrin, hepcidin, spag11, zap, dbi, cathelicidin, proenkaphalin, mbp, slpi, bpi) out of 22 AMPcg families. The statistical significance of NHR-related motif enrichment in this group is based on the Bonferroni corrected p-value obtained from the right-sided Fisher's exact test (corrected p-value = 1.237e-003) with the null-hypothesis that there is no enrichment of NHR. The second to fourth ranked groups include liver-specific TFs (ten families), adipocyte-specific TFs (eight families) and immune cell-specific TFs (five families).

Our computational study identified VDR as a potential controller of AMPcgs, but implied also other known, as well as new NHR candidates (Supplementary Table 6, Additional File 6). The computational methods we used produced a broader spectrum of AMP-regulating candidates than gene expression assays [32]. Therefore our study suggests that the influence of NHRs extends across multiple AMPcg families and beyond those already reported (Supplementary Table 3, Additional File 3).

### Other TFs and their potential role in AMPs

We also found several TFs that were frequently associated with genes of the 22 AMPcg families (Supplementary Table 6, Additional File 6). The binding motif for Sp1, an ubiquitous TF is enriched in the numerous GC-rich housekeeping gene promoters, but also contributes to tissue-specific transcription. For example, the Sp1 motif was detected in the promoters and enhancers of genes expressed in hematopoietic and epithelial cells where it appears to cooperate with lineage-restricted factors in directing their expression [37].

Meis1a and Meis1b isoforms are homeoproteins related to the pre-B cell transformation protein family. Meis1a is implicated in the myelopoesis [38] leading to the basophil, neutrophil and eosinophil granulocytes. We detected both Meis1a and Meis1b binding sites in members of the apoa2, calgranulin, slpi, granulin, secretogranin, mbp, vip, lysozyme AMPcg families, suggesting a granulocyte-specific transcriptional control function. Calvo and co-workers [38] showed that Meis1a suppressed the G-CSF-induced transcription of neutrophil differentiation-specific genes cytochrome b-245 beta, lactoferrin, early growth response-1, neutrophil gelatinase B, and lipopolysaccharide receptor CD14. The unique C-terminus of Meis1a which was shown to specifically mediate protein kinase A and trichostatin activation [39] provides additional support for the functional differences of Meis1a and Meis1b. Meis1a in combination with other neutrophil-specific TFs (i.e. STAT1, STAT6 and NF-kappa B) may play an important role in the recruitment and activation of neutrophils seen in sepsis and *Helicobacter pylori *infection-induced iron-deficiency [40,41]. Interestingly, hepcidin, which inhibits iron absorption from the small intestine during infection-induced inflammation, lacks Meis1, suggesting the induction of multiple alternative transcriptional regulation mechanisms during microbial pathogenesis.

### Promoter content of alpha-defensin, penk and zap families

For alpha-defensins, penk and zap family members we studied the predicted PEs and their positional arrangements in orthologs in detail to address questions of spatial differences in expression. In case of alpha-defensins and Penk, experimentally identified PEs were used to assess and interpret the predictions. For the zap family promoters with scarce experimental data our computational models suggested a co-involvement of Zap, NHRs and metal regulatory transcriptional control in innate immunity and oxidative stress.

### Alpha-defensin promoter model

Alpha-defensins are specific to mammals [42]. Gene duplication events probably led to both species-specific and functionally diverse subsets of alpha-defensins which should be also reflected in the upstream regulatory regions. For example, enteric-expressed defensins are important to the barrier function of the gut mucosal surface against bacteria, whereas myeloid and neutrophil-specific defensins help macrophages and neutrophils to kill internalized bacteria [43].

**Figure 2:**
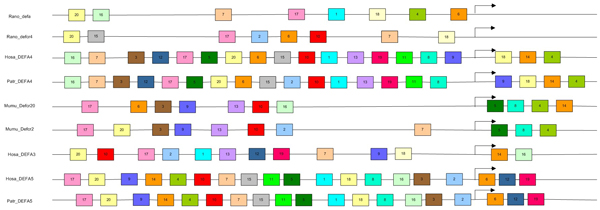
**Motif distribution in alpha-defensin promoters**. The boxes represent the *ab initio search-identitied *motifs. The numbers (i.e. 13) and colors refer to the different motifs. The black line connecting the boxes denotes the 1200 nt promoter region. The TSS is indicated by an arrow. The graphical representation of the motifs is not drawn to scale. The species abbreviations are Rano: *Rattus norvegicus*, Mumu: *Mus musculus*; Patr: *Pan troglodytes*; Hosa: *Homo sapiens*.

We were interested to investigate how the promoter content of rat, mouse and human alpha-defensins correlates with the enteric and myeloid/neutrophil cell expression. Human Defa3, chimpanzee Defa4, mouse Defa1 and rat Defa represent the myeloid-specific alpha-defensins that share the motif arrangement (17-1-18) in their promoter sequences (Fig. 2). The motif arrangement 17-1-18 means that we found motifs 17, 1 and 18 in this order in the examined promoters. Mouse defcr20, defcr2, rat defcr4, human and chimpanzee defa5 which share the motif organization 17-10-7 belong to the enteric-expressed group of alpha-defensins. Only motif 17 (GMASTTCTKT) which contains putative binding sites for IRF-1, IRF-3, NF-AT1, NF-AT2, NF-AT3 or NF-AT4 transcription factors occured in both categories (Supplementary Table 7, Additional File 7). Whether the motif is essential for the activation of alpha-defensin expression remains to be tested experimentally.

Interestingly, our comparison revealed also motif combinations that are common among myeloid and enteric defensins but largely different between rodents and primates. The rat defa and enteric-expressed mouse defcr2 promoter regions share the motifs 20 (AR PXR-1:RXR-alpha) – 7 (POU1F1a, POU2F1) – 4 (RAR-alpha1, RXR-alpha) (20-7-4) arrangement (Fig. 2, Supplementary Table 7, Additional File 7). In contrast, the primate myeloid-expressed (Hosa_DEFA4, Patr_DEFA4, Hosa_DEFA3) and enteric-expressed (Hosa_DEFA5 and Patr_DEFA5) alpha defensins share the motif organization (20-10-11-19).

**Figure 3:**
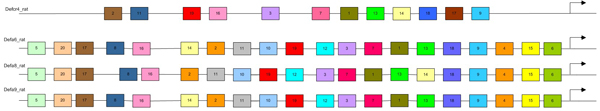
**Motif composition in the alpha defensin promoter regions of rat Defcr4, Defa6, Defa8 and Defa9**. Defa8 and Defa9 are the result of recent gene duplications events while Defcr4 is an ancient alpha defensin. The black line connecting the boxes denotes the 1200 nt promoter region. The TSS is indicated by an arrow. The graphical representation of the motifs is not drawn to scale.

Some AMP families such as defensins contain members that arose by gene duplication. Assuming differences in the promoter regions of recently duplicated defensins versus ancestral defensins we compared the upstream sequences (1000 nt) of four rat alpha defensins Defcr4, Defa6, Defa8 and Defa9. The latter three defensin genes are thought to be the result of gene duplications, while Defcr4 represents an ancestral alpha defensin [40]. We identified seven motifs (5, 6, 8, 10, 12, 15 and 20) that were common to Defa6, Defa8, Defa9 but not shared with Defcr4. On the other hand, motifs 1, 2, 3, 7, 9, 11, 13, 14, 16, 17, 18, 19 were identified in all four rat promoters. Fig. 3 and Supplementary Table 8, Additional File 8 show the distribution of the motifs across the rat promoter sequences and the corresponding TFBSs.

### Penk promoter model

PenkA is a neuropeptide-encoding gene which is primarily expressed in tissue cells of the mature nervous and neuroendocrine systems, the epididymis and in normal and activated lymphocytes [44]. Cleavage of PenkA results in the antibacterial peptide Enkelytin which is active against gram-positive bacteria for example, *Staphylococcus aureus *[45]. Penk-derived peptides have immunomodulatory properties ranging from increased natural killer cell cytotoxicity and monocyte chemotaxis to involvement in delayed-type hypersensitivity [44,46]. Our computational analysis of the promoter regions can provide some clues towards identifying PEs that confer the differences in immune and nervous tissue cell-specific expression.

**Figure 4:**
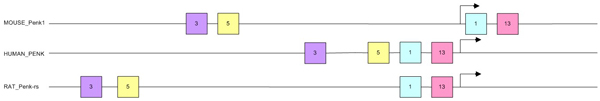
**Conserved penk motif organization in mouse, rat and human**. The numbers (i.e. 1) and colors refer to different motifs. The black line connecting the boxes denotes the 1200 nt promoter region. The TSS is indicated by an arrow. The graphical representation of the motifs is not drawn to scale.

The penk family promoter model derived from motifs (Supplementary Table 9A, Additional File 9) detected in mouse rat and human promoter sequences constitutes a single, conserved motif arrangement 3-5-1-13 (Fig. 4 and Supplementary Table 9B, Additional File 9). The candidate TFs (GR, AR (motif3), NF-kappaB, AP-2 (motif5), RXR-alpha, LXR-alpha, ERRalpha1 (motif 1)and DSF, GCN4, COUP-TF1, RAR-beta, RXR-alpha, RAR-alpha1, TLX, Pax-2.1 (motif 13) that may bind these motifs are probably necessary for expression but not sufficient to confer differential spatial expression. For instance, the expression of penk in the epididymis is regulated by testicular factors that control expression via members of the Ets transcription family [47]. Motif 7 contains submotifs identical to binding sites of the Ets family transcription factors c-Ets1, Elk-1, SAP-1a, SAP-1b, PEA3 and ELF-1. In analogy, motif 12 contains binding sites for USF family transcription factors which contribute to the transcriptional regulation of calcium-inducible neuronal genes [48,49].

### Zap promoter model

**Figure 5:**
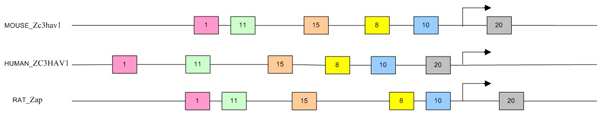
**Conserved Zap motif organization in mouse, human and rat**. The numbers (i.e. 1) and colors refer to different motifs. The black line connecting the boxes denotes the 1200 nt promoter region. The TSS is indicated by an arrow. The graphical representation of the motifs is not drawn to scale.

The CCCH-type zinc finger protein family member Zap acts as an antiviral protein against Sindbis and Moloney murine leukemia virus [50]. Its antiviral activity is mediated through the disruption of viral messenger RNAs in the cytoplasm without affecting the levels of nuclear mRNA [51]. The Zap promoter region contains twenty motifs (Fig. 5 and Supplementary Table 10A, Additional File 10) including eight potential NHR binding motifs (1, 2, 5, 6, 8, 9 and 14). The motif organization 1-11-15-8-10-20 is conserved (Fig. 5 and Supplementary Table 10B, Additional File 10) and potentially associated with two unknown TFs (motifs 10 and 15), Alfin1, RXR-alpha, VDR, E12, E47, MyoD, myogenin, EMF1, EMF2, EMF3, EMF4, Myf-5, c-Myc, USF2, CAN, E2A, DEP2, HEB, Ac, AS-C T3, Da, Sc, Sn, CLIM2, GATA-1, Lmo2, Tal-1, USF-1, NeuroD, NEUROD, LVa, PR B, AR, GR, c-Ets-2, ESE-1, HELIOS, LyF-1 (motif1), NF-1, TGGCA-binding protein (motif11), LyF-1, RXR-beta, VDR (motif8) and MTF-1 (motif20). The presence of NF-1 TFBS in both penk and zap families, suggests that transcripts of these families might be induced by steroid hormones that interact with NF-1 [52]. Zap expression levels in liver and kidney are high. The presence of a putative binding site for metal-regulatory transcription factor MTF-1, which is also expressed in liver and kidney, suggests a regulatory role of Zap in heavy metal load, oxidative stress, hypoxia and innate immunity [53,54].

### Suggested future experiments

Our analysis generated a number of hypotheses that are in good concordance with some of the existing knowledge in the field. However, the computationally-inferred hypotheses can only be tested in experiments. For example, microarray technology combined with chromatin immunoprecipitation (ChIP) profiling [55] can be used to identify all the chromosomal locations that are occupied by a transcription factor. These experiments are expected to clarify which promoters and TFs are specific for certain tissue cells and how many AMPcgs are regulated by a TF, TF pairs or multiple TFs. Eventually, the combination of both computational and experimental approaches should permit us to construct mechanistic models of AMPcg regulatory transcription networks.

## Conclusion

The large-scale computational analysis of promoters derived from 22 families of AMPcgs across three mammalian species has allowed us to identify potential key transcription elements of these families. We have analyzed [-1000, +200] promoter regions and it is likely that we may have missed out regulatory elements farther upstream that might be important in the fine-tuning of the regulation of particular families of AMPcg. Our analysis showed that TFs of the liver-, nervous system- specific and NHR groups were overrepresented in promoters of AMPcg families. These TF groups consist of transcription regulators that are involved in diverse physiological functions, including the control of embryonic development, cell differentiation and homeostasis, but also in immune response. Interestingly, NHRs were more prominent than immune cell-specific TFs in the analyzed AMPcg families. Experimental evidence showed the involvement of NHRs in various immunomodulatory pathways [56-58]. However little is known about their direct involvement in innate immunity. Recently, there has been evidence that VDR plays a direct role in the induction of antimicrobial innate immune response [59]. The results of the computational analysis which took a bird's eye view of the transcriptional regulators involved in multiple AMPcg families, concur with this evidence and revealed a number nuclear hormone receptor as candidates. For example, GR, RXR-alpha, AR, VDR and T3R-alpha, seem to be involved in control of 20, 18, 17, 16 and 15 families respectively, out of 22 analyzed. In addition we discovered 102 new motifs as candidate TFBS with a role in antimicrobial innate immunity. The actual experimental confirmation of the AMPcg transcription regulatory elements can only be accomplished by targeted research of infection or cellular stress models using time-course sampled tissue cell types.

## Methods

The overall methodology is schematically depicted in Fig. 1. AMP sequences were extracted from the ANTIMIC database [8,60] that contains 1439 non-redundant AMPs and GenBank [61]. We used TBLASTN [13] with BLOSUM45 matrix to search 102,801 flcDNAs of the FANTOM collection [11] (FANTOM1+2 (60,770) plus FANTOM3 (42,031)) against AMP protein sequences of ANTIMIC. Since TBLASTN translates the query sequence into six possible open-reading frames, cDNAs with short CDS below the protein-coding annotation threshold can be captured. Of 183 mouse translated flcDNAs with sequence identities equal or greater than 60% to known AMPs over the length of 100 residues and with E-value of 0.01 or less, five were identified as false positives by checking their stable gene name and gene ontology annotations. Less stringent threshold settings (i.e. 50% or 55%) applied to a test set of cathelicidins, alpha and beta defenins resulted in to too many false positives (data not shown) without gaining any new AMPcg candidates among the FANTOM sequence set.

### Extraction of promoter regions

The mouse flcDNA were annotated with their official gene names and symbols, associated representative cDNAs, chromosomal localization information, TUID (transcriptional unit ID) and CAGE TSS (transcription start site information based on CAGE tags) [6]. We then determined for the AMP-coding mouse flcDNAs the human and rat orthologs using HomoloGene [61] and Entrez Gene [62]. In our analysis we did not include all the members of a particular AMP family but only those members that were captured in BLAST searches against the FANTOM3 mouse cDNAs. In addition, each of these ortholog groups was manually checked for synteny. The promoter regions of the orthologs in human and rat were extracted using PromoSer [63,64] and FIE2 [65,66] programs, as well as H-Invitational database [67]. All three resources provided estimated transcription start site (TSS) locations based on mapping EST and flcDNA sequences to genomic sequences. The extracted mouse, human and rat promoter regions covered [-1000, +200] relative to the estimated transcription start site location. In the case of multiple TSS locations in human and rat sequences we extracted the most 5' one. The TSS location of mouse sequences was determined by using the start position of the first exon of the FANTOM cDNA-genome mapping data [68]. Mouse promoter sequences [-1000, +200] were then extracted by mapping the TSS location to the mouse genome assembly from the UCSC Genome Browser [69]. Our final dataset contained 77 mouse, rat and human promoters. Only seven mouse sequences had associated CAGE tag information (Supplementary Table 2, Additional File 2). Therefore, we estimated the TSS location for all sequences based on the 5'end of the flcDNA data. For histone2a genes we extracted a region of [-200, +100] relative to the TSS because these genes appeared to have bidirectional promoters within 200 nucleotides (nt) of the TSS.

### Motif search

The promoter sequences were submitted to the Dragon Motif Builder (DMB) program [21,70] for *ab initio *motif finding. The Expectation Maximization (EM) threshold was set to 0.85 for all families that lacked experimentally confirmed TF binding sites (TFBSs) in their promoters. One should note that there is no rule about what is the optimal threshold. In fact, the optimal threshold is likely to be different for different promoter sets. Thus, we used the somewhat arbitrary threshold of 0.85 because it resulted in relatively specific matrix families. Since the algorithm is heuristic, different thresholds usually produce different results. In the cases of known functional TFBSs for a AMpcg family we used two different thresholds (0.85, 0.75) and selected the one that fitted better to the experimentally confirmed TFBSs. The program was set to search for 20 motif families, with motifs of length 10 to 15 nt within each of the 22 AMPcg families. In total we identified 440 motif families. In the case of histone2a family we chose a shorter motif length of 8–12 nt because the promoter length of histone2a family was shorter than for the other families. After DMB identified the sequence motifs, we used the Patch program (mismatch = 0; motif length = 6; species =all) [17] of TRANSFAC professional database ver. 8.4 to infer potential transcription factors (TFs) that may bind to motifs of these families. Promoter models were created from motifs that were conserved among all promoter sequences of the analyzed AMPcg family.

To find motif families that are common across multiple AMPcg families, we combined all 440 motifs and searched the most commonly found sub-motif families in them. We used the DMB program and searched for motifs of 6–8 nt length. The reduction of motif length did not cause over-prediction of motifs since the search was restricted to sequences of the previously identified motifs of length 10–15 nt. Potential motif-binding TFs were identified by the Patch program as already described. It was possible to extract common motifs in different AMPcg families using simultaneously promoters from all families. However, this would bias the result as it will 'enforce' finding common motifs. We adopted a less biased strategy of identifying the motifs independently for each family and then identifying commonalities across various AMPcg families.

In case of the penk family, we used three programs DMB, MEME [22,71] and Improbiser [23] to search for motifs of 10–15 nt length. All three programs utilize the EM algorithm. Improbiser can identify a maximum of six motif families. For MEME and DMB we identified 20 motif families and selected the top six families based on e-value. This threshold setting allowed us to obtain comparable results from three different programs and to select the most appropriate one for our data analyses. The motifs were then compared with TRANSFAC database entries to obtain TFs that can potentially bind to these motifs.

### Generation of motif models

We searched for all possible combinations of motifs that were present in identical strand-orientation and order and constrained by a defined range of distances between the motifs for a given set of promoter sequences. A Perl program was used to extract the motif arrangement models from the graphic motif representation file generated by DMB. This program requires as user input the maximum allowed distance between two successive motifs expressed as percentage of the total promoter length and a numeric value for the minimum number of motifs. Promoter models that contained motifs within the specified constraints were selected. If more than one model was possible the model with the maximum number of motifs was selected. If some of the multiple possible models contained motifs that corresponded to experimentally proven TFBSs for the considered AMP gene family, then these models were selected. The minimum number of motifs per model was set to three. The distance constraint was tested for the interval of 1%-30% of the total promoter length (1200 nt). It was observed that promoter models having three or more motifs could be generated with distance percentages of 20% to 30%. This distance percentage appeared to be suitable for the promoter length of 1200 nt that we used. Hence, the distance between two adjacent motifs in a promoter model ranges between one to 240 nt or up to 300 nt. The motif combinations that appeared common across all promoters of a given AMP family were chosen as candidates for scanning the promoter dataset.

### Statistical significance of potential NHR-binding motifs

Since we observed that many AMPcg families have in their promoters a significant proportion of motifs that potentially bind NHRs, we focussed on finding out what group of AMPcg families is the most enriched in such motifs, and to see if this enrichment is statistically significant. This is a non-trivial problem for example, it is obvious that only collections of the AMPcg families that are most enriched in these motifs should appear in the group. However, we do not know how much they should be enriched individually to become members of that group. In addition, the enrichment of individual AMPcg families in these motifs could be statistically insignificant, but still the group of families could have statistically significant enrichment. Also, even if we find the most enriched group, there is no guarantee that the enrichment will be statistically significant. To solve the problem we applied the following procedure. All families were sorted by the number of motifs that may bind NHR. Then we split AMPcg families into two groups A and B. In group B we included the family that had the least number of such motifs. The remaining families were placed in group A. We calculated the p-value for the enrichment in motifs that may bind NHR. The p-value was determined using the hypergeometric distribution and the right-side Fisher's exact test and was corrected by the Bonferroni method for the 440 tests (this is the number of motifs families identified; 20 motif families for each of the 22 AMPcg families). We then excluded from group A the AMPcg family with the next least number of target motifs and added that family to group B. We repeated the calculation of the p-value. The process of eliminating AMPcg families from group A is repeated until A contained the last of the 22 AMPcg families. Based on the 21 p-values calculated this way (Supplementary Table 11, Additional File 11), we determined the one with the smallest value, 2.81167E-06 (Bonferroni corrected value = 0.001237134) which belongs to the group of 11 NHR binding motif enriched AMPcg families.

## List of Abbreviations

AMP: antimicrobial peptide; TFBS: transcription factor binding site; PE: promoter element; AMPcgs: AMP coding genes; apoa2: (apolipoproteinA-II); mbp: myelin basic protein; Slpi: skin-derived antileukoproteinase; SPYY: skin peptide tyrosine-tyrosine; vip: vasoactive intestinal peptide; zap: CCCH type, antiviral 1 protein; penk1: proenkaphalin1(penk A); DMB: Dragon Motif Builder; TF: transcription factor; flcDNA: full-length cDNA; nt: nucleotide(s); IMR: immune related; NHR: nuclear hormone receptor.

## Authors' contributions

MB, CS and VBB conceived the study. MB, LY, EH, SLT, RC, SPTK and VBB performed computational studies. MB, CS, VBB analyzed data. MB, CS, CYL, DAH and VBB wrote the manuscript. CK, JK, PC, YH contributed data for experiments.

## Supplementary Material

Additional file 2 Supplementary table 2. AMPeg families and representative members in mouse, rat and human. Click here for file

Additional file 3 Supplementary table 3. TFs associated with *ab initio*-predicted TFBSs that coincided with experimental data. Click here for file

Additional file 4 Supplementary table 4. Over-represented motifs that are common in multiple AMP families. Click here for file

Additional file 5 Supplementary tables 5A and 5B Distribution of motifs corresponding to different TF groups among AMP families and Ranking of TF groups according to their frequency of appearance in different AMP families. Click here for file

Additional file 6 Supplementary table 6. Distribution of individual transcription factors among AMP families. Click here for file

Additional file 7 Supplementary table 7. Common motifs detected among groups of enteris and myeloid-specific alpha-defensins. Click here for file

Additional file 8 Supplementary table 8. Motif distribution across the rat *Defcr4*, *Defa6*, *Defa8 *and *Defa9 *promoter regions. Click here for file

Additional file 9 Supplementary tables 9A and 9B. TF binding sites that correspond to *ab initio*-predicted motifs derived from the Penk family promoter regions and Motif arrangements in promoter region in mouse (4922504O09), human (HIX0007519.2) and rat (NM_017139) of Penk family members. Click here for file

Additional file 10 Supplementary tables 10A and 10B. TF binding sites that correspond to *ab initio*-predicted motifs derived frokm Zap family promoter regions and Promoter motif arrangements in mouse (FA20004O17), human (HIX0007129.3) and rat (NM_173045) Zap family members. Click here for file

Additional file 11 Supplementary table 11. P-value table of motif groups. Click here for file

Additional file 1 Supplementary table 1. FANTOM3 dataset-derived AMP transcripts which were new to mouse and absent in human. Click here for file

## Figures and Tables

**Table 1:** Total number of motifs found in each AMP family

AMP family	New AMPcg- associated TFs	Unknown motifs	Total
Alpha-defensin	73	3	77
Apoa2	36	6	42
BPI	113	4	120
Beta-defensin	78	8	89
Spag11	75	3	78
Calgranulin	162	4	166
Cathelicidin	75	3	81
DBI	53	1	59
Granulin	67	3	70
Hepcidin	59	3	63
Histone 2A	83	12	97
Lactoferrin	46	4	52
Lysozyme	30	9	41
MBP	67	2	70
Melanotropin alpha	81	1	84
Proenkaphalin1	54	3	85
SPYY	58	5	63
Secretogranin I	31	6	39
Slpi	94	5	101
VIP	54	3	60
Vasostatin	19	9	30
ZAP	77	5	82

**Table 2:** Distribution of different tissue/function-specific TF groups among AMP families

	Tissue/function-specific TF groups												Cut-Off [%]
		
	AD	NHR	CC	IMM	LIV	LUNG	MUS	NS	PAN	PIT	EYE	BS	
Total no. of motifs	131	139	97	122	141	122	78	143	77	74	1	12	
No. of AMPcg families	17	18	8	14	19	16	4	17	5	5	0	0	25
	14	12	7	11	14	10	4	15	3	2	0	0	30
	8	11	3	6	10	5	2	11	2	1	0	0	35
	4	7	3	4	3	4	1	9	0	0	0	0	40

Tissue/function-specific TF groups are AD: adipocyte-related TFs; NHR: nuclear hormone receptor TFs; CC: cell cycle-related TFs; IMM: immune cell-specific TFs; LIV: liver cell-specific TFs; LUNG: lung cell-specific TFs; MUS: muscle cell-specific TFs; NS: nervous system-related TFs; PAN: pancreatic B-cell related; PIT: pituitary gland-specific TFs; Eye: eye-specific TFs; BS: bone-specific TFs. TF that groups (AD, NHR etc.) occur with highest frequency among AMP families are underlined. Cut-off indicates the minimum percentage of motifs in a TF family that can bind TFs from a particular tissue/function-specific group.
