# Supplementary material for: Computational promoter analysis of mouse, rat and human antimicrobial peptide-coding genes
Source: BMC Bioinformatics. 2006 Dec 18;7(Suppl 5):S8. doi: 10.1186/1471-2105-7-S5-S8 (PMC1764486; doi:10.1186/1471-2105-7-S5-S8)
Supplement: Additional file 2 — Supplementary table 2. AMPeg families and representative members in mouse, rat and human. [file 1471-2105-7-S5-S8-S2.pdf]

Supplementary Table 2. AMPcg families and representative members in mouse, rat and human. Mm: *Mus musculus*; Hs: *Homo sapiens*; Rn: *Rattus norvegicus*: TUID: transcriptional unit ID; CTSS: transcription start site (TSS) information based on CAGE tags.

AMP Family	Gene symbol	Species	Representative CloneID/Accession	TUID	CTSS
Alpha defensin	2010016B13Rik	Mm	2010016B13	175722	No
	2010016F14Rik	Mm	2010016F14	168136	No
	DEFA5	Hs	NM_021010	-	-
	DEFA6	Hs	NM_001926	-	-
	DEFA4	Hs	NM_001925	-	-
	DEFA3	Hs	NM_005217	-	-
Apoa2 (apolipoprotein A-II)	Apoa2	Mm	I530003A11	83109	No
	APOA2	Hs	HIT000032344.2	-	-
	Apoa2	Rn	NM_013112	-	-
Beta defensin	9230107O10Rik	Mm	9230107O10	103672	No
	DEFB28	Hs	AF525930	-	-
	Defb1	Mm	D630029A12	169116	Yes
	DEFB1	Hs	BC033298	-	-
	Defb1	Rn	NM_031810	-	-
	Defb23	Mm	1700012K18	121132	Yes

	Defb123	Hs	NM_153324	-	-
	Defb4	Mm	2310001F05	168175	No
	DEFB4	Hs	NM_004942	-	-
	Defb36	Mm	1700011J22	168985	Yes
	DEFB105a	Hs	NM_152250	-	-
	Defb12	Mm	9230103N16	77756	No
	Defb19	Mm	4930563B01	81337	No
BPI (Bactericidal/permeability-increasing)	9230105K17Rik	Mm	9230105K17	112251	No
	BPI	Hs	BC040955	-	-
Bin1b/SPAG11	Spag11	Mm	9230111C08	168760	No
	SPAG11	Hs	NM_016512	-	-
	Spag11	Rn	NM_145087	-	-
Cathelicidin	Camp	Mm	F930015N03	112000	Yes
	CAMP	Hs	NM_004345	-	-
	cramp	Rn	AF484553	-	-
Calgranulin	S100a9	Mm	F430201H11	83114	Yes
	S100a9	Hs	NM_002965	-	-
	S100a9	Rn	NM_053587	-	-

DBI (Acyl-CoA-binding protein family)	Dbi	Mm	6720460E16	102356	Yes
	DBI	Hs	NM_020548	-	-
Slpi (skin-derived antileukoproteinase)	Slpi	Mm	2310075E18	75903	No
	SLPI	Hs	HIT000038907.2	-	-
	Slpi	Rn	NM_053372	-	-
Granulin	Grn	Mm	0610012H06	104193	Yes
	GRN	Hs	BC000324	-	-
	Grn	Rn	NM_017113	-	-
Hepcidin	1810073K19Rik	Mm	2210420P15	168118	Yes
	LEAP2	Hs	NM_052971	-	-
Histone 2A derived defense peptide	Hist1h2ac	Mm	9030420B16	112273	No
	HIST1H2AC	Hs	NM_003512	-	-
	Hist1h2ae	Mm	1190022L06	112736	No
	HIST1H2AE	Hs	NM_021052	-	-
Lactoferrin	Ltf	Mm	9830118D19	173811	No
	LTF	Hs	NM_002343	-	-
Lysozyme	9530003J23Rik	Mm	9530003J23	106239	No

	Lyzs	Mm	I420013M05	111075	Yes
	LYZS	Hs	AF099029	-	-
	Lyzs	Rn	NM_012771	-	-
MBP (Myelin Basic Protein)	Prg2	Mm	2510004C07	112877	No
	PRG2	Hs	HIX0009634.2	-	-
	prg2	Rn	NM_031619	-	-
Melanotropin alpha (Pro-opiomelanocortin family)	Pomc1	Mm	5730403F20	151196	No
	POMC1	Hs	NM_000939	-	-
	Pomc1	Rn	NM_139326	-	-
PENK (Proenkaphalin) (opioid neuropeptide family)	Penk1	Mm	4922504O09	179452	Yes
	PENK	Hs	HIX0007519.2	-	-
	Penk-rs	Rn	NM_017139	-	-
Secretogranin I (chromogranin/secretogranin family)	Chgb	Mm	5730420J08	177050	Yes
	CHGB	Hs	HIX0015625.2	-	-
	Chgb	Rn	NM_012526	-	-

SPYY (Skin peptide tyrosine-tyrosine) (NPY family)	Npy	Mm	0710005A05	72959	Yes
	Pyy	Mm	C820007C10	111251	Yes
	NPY	Hs	HIX0006525.2	-	-
	Npy	Rn	NM_012614	-	-
Vasostatin (Chromogranin A) (chromogranin/secretogranin family)	Chga	Mm	G630083O06	83089	Yes
	CHGA	Hs	HIX0011909.2	-	-
	Chga	Rn	NM_021655	-	-
VIP (Vasoactive intestinal peptide) (Glucagon family)	Vip	Mm	9130007F05	112113	No
	VIP	Hs	HIX0006306.2	-	-
ZAP (CCCH type, antiviral 1)	Zc3hav1	Mm	F420004O17	99218	Yes
	ZC3HAV1	Hs	HIX0007129.3	-	-
	Zap	Rn	NM_173045	-	-
