# Supplementary material for: Computational promoter analysis of mouse, rat and human antimicrobial peptide-coding genes
Source: BMC Bioinformatics. 2006 Dec 18;7(Suppl 5):S8. doi: 10.1186/1471-2105-7-S5-S8 (PMC1764486; doi:10.1186/1471-2105-7-S5-S8)
Supplement: Additional file 3 — Supplementary table 3. TFs associated with ab initio-predicted TFBSs that coincided with experimental data. [file 1471-2105-7-S5-S8-S3.pdf]

Supplementary Table 3. TFs associated with *ab initio*-predicted TFBSs that coincided with experimental data

AMP family	Experimentally determined TFs	References	Predicted TFs matching experimental confirmed TFs
Alpha defensin	CAAT, PEBP2/CBF	[1]	PEBP2/CBF
Beta defensin	NF-KAPPAB, AP-1, NF-IL6, MEF, VDR	[2],[3],[4], [5]	AP-1,MEF(C-ETS1),VDR
BPI	AML-1, PU.1, SP3/SP1,C/EBP,USF, NF-KB, C-REL	[6]	SP3/SP1,AML-1,NF-KB
Cathelicidin	VDR, NF-IL6, RAR,IL-6RE	[5], [7], [8]	VDR, RAR, IL-6 RE
DBI	SREBP, SP1, PPAR-ALPHA,AP-1, C/EBP, HNF-3, RXR-ALPHA, NF-1/CTF , AP-2	[9], [10]	SREBP, SP1,AP-1,RXR-ALPHA,NF-1/CTF
Hepcidin	C/EBP-ALPHA	[11]	C/EBPALPHA
Histone 2A	TBP,OCT-1,CAAT box,	[12], [13], [14]	Oct-1, CAAT
Lactoferrin	SP1, C/EBP	[15], [16]	SP1, C/EBP
Lysozyme	SP1,MEF,C/EBP	[17]	MEF (C-ETS1),SP-1
MBP	NKX2.2,SP1,SOX10,PAX3, NF-KB	[18], [19], [20], [21], [22]	SP1
Melanotropin-alpha	SRE,AP-1, AP-2 LIKE, CAAT BOX	[23]	AP-1, AP-2 LIKE
Proenkaphalin1	TATA, AP-2, NF-KAPPAB,MZF-1,MYC PACH1,CREB,CRE, NF1,AP-1	[24], [25], [26] [27]	AP-1, NF1, TATA,AP2,NF-KB,MZF-1,NF-Y,
Secretogranin I	SP-1, CRE, TATA	[28], [29]	SP-1, TATA
Vasostatin	OLF/EBF, SP1,CREB,GR	[30], [31], [32], [33]	GR,SP-1

VIP	OCT-1,MEF-2,STAT,AP-1,CRE	[34]	STAT1,AP-1,POUF1A(OCT-1)
Slpi	GR,PR, IRF-1	[35], [36], [37]	GR, PR
Apoa2	NA	NA	NA
Calgranulin	NA	NA	NA
Granulin	NA	NA	NA
SPYY	NA	NA	NA
ZAP	NA	NA	NA
Bin1b/SPAG11	NA	NA	NA

References

1. Yamamoto CM, Banaiee N, Yount NY, Patel B, Selsted ME. **Alpha-defensin expression during myelopoiesis: identification of cis and trans elements that regulate expression of NP-3 in rat promyelocytes**. *J Leukoc Biol* 2004, **75**: 332-341.
2. Harder J, Meyer-Hoffert U, Teran LM, Schwichtenberg L, Bartels J, et al. **Mucoid Pseudomonas aeruginosa, TNF-alpha, and IL-1beta, but not IL-6, induce human beta-defensin-2 in respiratory epithelia**. *Am J Respir Cell Mol Biol* 2000, **22**: 714-721.
3. Vora P, Youdim A, Thomas LS, Fukata M, Tesfay SY, et al. **Beta-defensin-2 expression is regulated by TLR signaling in intestinal epithelial cells**. *J Immunol* 2004, **173**: 5398-5405.
4. Lu Z, Kim KA, Suico MA, Shuto T, Li JD, et al. MEF up-regulates human beta-defensin 2 expression in epithelial cells. *FEBS Lett* 2004, **561**: 117-121.
5. Wang TT, Nestel FP, Bourdeau V, Nagai Y, Wang Q, et al. **Cutting edge: 1,25-dihydroxyvitamin D3 is a direct inducer of antimicrobial peptide gene expression**. *J Immunol* 2004, **173**: 2909-2912.
6. Lennartsson A, Pieters K, Ullmark T, Vidovic K, Gullberg U. **AML-1, PU.1, and Sp3 regulate expression of human bactericidal/permeability-increasing protein**. *Biochem Biophys Res Commun* 2003, **311**: 853-863.

7. Frohm Nilsson M, Sandstedt B, Sorensen O, Weber G, Borregaard N, et al. **The human cationic antimicrobial protein (hCAP18), a peptide antibiotic, is widely expressed in human squamous epithelia and colocalizes with interleukin-6**. *Infect Immun* 1999, **67**: 2561-2566.
8. Wu H, Zhang G, Minton JE, Ross CR, Blecha F. **Regulation of cathelicidin gene expression: induction by lipopolysaccharide, interleukin-6, retinoic acid, and Salmonella enterica serovar typhimurium infection**. *Infect Immun* 2000, **68**: 5552-5558.
9. Sandberg MB, Bloksgaard M, Duran-Sandoval D, Duval C, Staels B, et al. **The gene encoding acyl-CoA-binding protein is subject to metabolic regulation by both sterol regulatory element-binding protein and peroxisome proliferator-activated receptor alpha in hepatocytes**. *J Biol Chem* 2005, **280**: 5258-5266.
10. Elholm M, Bjerking G, Knudsen J, Kristiansen K and Mandrup S. **Regulatory elements in the promoter region of the rat gene encoding the acyl-CoA-binding protein**. *Gene* 1996, **173**: 233-238.
11. Courselaud B, Pigeon C, Inoue Y, Inoue J, Gonzalez FJ, et al. **C/EBPalpha regulates hepatic transcription of hepcidin, an antimicrobial peptide and regulator of iron metabolism. Cross-talk between C/EBP pathway and iron metabolism**. *J Biol Chem* 2002, **277**: 41163-41170.
12. Oswald F, Dobner T, Lipp M. **The E2F transcription factor activates a replication-dependent human H2A gene in early S phase of the cell cycle.** *Mol Cell Biol* 1996, **16**: 1889-1895.
13. Albig W, Trappe R, Kardalinou E, Eick S, Doenecke D. **The human H2A and H2B histone gene complement.** *Biol Chem* 1999, **380**: 7-18.
14. Trappe R, Doenecke D, Albig W. **The expression of human H2A-H2B histone gene pairs is regulated by multiple sequence elements in their joint promoters**. *Biochim Biophys Acta* 1999, **1446**: 341-351.
15. Teng CT. **Lactoferrin gene expression and regulation: an overview**. *Biochem Cell Biol* 2002, **80**: 7-16.
16. Khanna-Gupta A, Zibello T, Simkevich C, Rosmarin AG, Berliner N. **Sp1 and C/EBP are necessary to activate the lactoferrin gene promoter during myeloid differentiation**. *Blood* 2002, **95**: 3734-3741.
17. Suico MA, Koga T, Shuto T, Hisatsune A, Lu Z, et al. **Sp1 is involved in the transcriptional activation of lysozyme in epithelial cells**. *Biochem Biophys Res Commun* 2004, **324**: 1302-1308.
18. Wei Q, Miskimins WK, Miskimins R. **Stage-specific expression of myelin basic protein in oligodendrocytes involves Nkx2.2-mediated repression that is relieved by the Sp1 transcription factor**. *J Biol Chem* 2005, **280**: 16284-16294.

19. Wei Q, Miskimins WK, Miskimins R.. *J Neurosci Re* **Sox10 acts as a tissue-specific transcription factor enhancing activation of the myelin basic protein gene promoter by p27Kip1 and Sp1***s* 2004, **78**: 796-802.
20. Wei Q, Miskimins WK, Miskimins R. **Cloning and characterization of the rat myelin basic protein gene promoter**. *Gene* 2003, **313**: 161-167.
21. Slutsky SG, Kamaraju AK, Levy AM, Chebath J, Revel M. **Activation of myelin genes during transdifferentiation from melanoma to glial cell phenotype**. *J Biol Chem* 2003, **278**: 8960-8968.
22. Huang CJ, Nazarian R, Lee J, Zhao PM, Espinosa-Jeffrey A, et al. **Tumor necrosis factor modulates transcription of myelin basic protein gene through nuclear factor kappa B in a human oligodendroglioma cell line**. *Int J Dev Neurosci* 2002, **20**: 289-296.
23. Deen PM, Terwel D, Bussemakers MJ, Roubos EW, Martens GJ. **Structural analysis of the entire proopiomelanocortin gene of Xenopus laevis**. *Eur J Biochem* 1991, **201**: 129-137.
24. Liu F, Kondova I, Kilpatrick DL. **Detection of PACH1, a nuclear factor implicated in the transcriptional regulation of meiotic and early haploid stages of spermatogenesis**. *Mol Reprod Dev* 2000, **57**: 224-231.
25. Kobierski LA, Wong AE, Srivastava S, Borsook D, Hyman SE. **Cyclic AMP-dependent activation of the proenkephalin gene requires phosphorylation of CREB at serine-133 and a Src-related kinase**. *J Neurochem* 1999, **73**: 129-138.
26. Fu W, Shah SR, Jiang H, Hilt DC, Dave HP, et al. **Transactivation of proenkephalin gene by HTLV-1 tax1 protein in glial cells: involvement of Fos/Jun complex at an AP-1 element in the proenkephalin gene promoter**. *J Neurovirol* 1997, **3**: 16-27.
27. Le Y, Gagneten S, Larson T, Santha E, Dobi A, et al. Far-upstream elements are dispensable for tissue-specific proenkephalin expression using a Cre-mediated knock-in strategy. *J Neurochem* 2003, **84**: 689-697.
28. Pohl TM, Phillips E, Song KY, Gerdes HH, Huttner WB, et al. **The organisation of the mouse chromogranin B (secretogranin I) gene**. *FEBS Lett* 1990, **262**: 219-224.
29. Mahata SK, Mahapatra NR, Mahata M, O'Connor DT. **Neuroendocrine cell type-specific and inducible expression of chromogranin/secretogranin genes: crucial promoter motifs**. *Ann N Y Acad Sci* 2002, **971**: 27-38.
30. Persson P, Manetopoulos C, Lagergren A, Nygren J, Gisler R, et al. **Olf/EBF proteins are expressed in neuroblastoma cells: potential regulators of the Chromogranin A and SCG10 promoters**. *Int J Cancer* 2004, **110**: 22-30.

31. Mahapatra NR, Mahata M, O'Connor DT, Mahata SK. **Secretin activation of chromogranin A gene transcription. Identification of the signaling pathways in cis and in trans**. *J Biol Chem* 2003, **278**: 19986-19994.
32. Hocker M, Raychowdhury R, Plath T, Wu H, O'Connor DT, et al. **Sp1 and CREB mediate gastrin-dependent regulation of chromogranin A promoter activity in gastric carcinoma cells**. *J Biol Chem* 1998, **273**: 34000-34007.
33. Rozansky DJ, Wu H, Tang K, Parmer RJ, O'Connor DT. **Glucocorticoid activation of chromogranin A gene expression. Identification and characterization of a novel glucocorticoid response element**. *J Clin Invest* 1994, **94**: 2357-2368.
34. Hahm SH, Eiden LE. **Cis-regulatory elements controlling basal and inducible VIP gene transcription**. *Ann N Y Acad Sci* 1998, **865**: 10-26.
35. Hayashi R, Wada H, Ito K, Adcock IM. **Effects of glucocorticoids on gene transcription**. *Eur J Pharmacol* 2004, **500**: 51-62.
36. King AE, Morgan K, Sallenave JM, Kelly RW. **Differential regulation of secretory leukocyte protease inhibitor and elafin by progesterone**. *Biochem Biophys Res Commun* 2003, **310**: 594-599.
37. Nguyen H, Teskey L, Lin R, Hiscott J. **Identification of the secretory leukocyte protease inhibitor (SLPI) as a target of IRF-1 regulation**. *Oncogene* 1999, **18**: 5455-5463.
