# Supplementary material for: Computational promoter analysis of mouse, rat and human antimicrobial peptide-coding genes
Source: BMC Bioinformatics. 2006 Dec 18;7(Suppl 5):S8. doi: 10.1186/1471-2105-7-S5-S8 (PMC1764486; doi:10.1186/1471-2105-7-S5-S8)
Supplement: Additional file 4 — Supplementary table 4. Over-represented motifs that are common in multiple AMP families. [file 1471-2105-7-S5-S8-S4.pdf]

Supplementary Table 4. Over-represented motifs that are common in multiple AMP families. Total AMP: The number of AMP families that contain the motif; Seq IDs: The mRNA ids of AMPcgs whose promoter sequences are analyzed. If the motif was detected in mouse (Mm), rat (Rn) and/or human (Hs) it is denoted by “+” else, by “-“.

No.	Pattern	TF name	Total AMP	AMP Family	Seq IDs	Mm	Hs	Rn
1	ACAGCA	GR	10	Alpha defensin	2010016B13, 2010016F14, NM_021010, NM_001926, NM_001925, NM_005217	+	+	-
				Apoa2	I530003A11, HIT000032344.2, NM_013112	+	+	+
				BPI	9230105K17, BC040955	+	+	-
				Calgranulin	F430201H11, NM_002965, NM_053587	+	+	+
				Hepcidin	NM_052971, 2210420P15	+	+	-
				Histone 2A	9030420B16, NM_003512, 1190022L06, NM_021052	+	+	-
				Melanotropin alpha	5730403F20, NM_000939, NM_139326	+	+	+
				Secretogranin	5730420J08, HIX0015625.2, NM_012526	+	+	+
				Vasostatin	G630083O06, HIX0011909.2, NM_021655	+	+	+
				ZAP	F420004O17, HIX0007129.3, NM_173045	+	+	+
2	AGGAAA	PEA3<br>c-Ets1<br>E74A<br>PU.1<br>LyF-1<br>c-Ets-2<br>ISGF-3<br>NF-AT<br>NF-AT2N<br>F-AT4<br>DEAF-1	11	Alpha defensin	2010016B13, 2010016F14, NM_021010, NM_001926, NM_001925, NM_005217	+	+	-
				BPI	9230105K17, BC040955	+	+	-
				Calgranulin	F430201H11, NM_002965,	+	+	+

					NM_053587			
				Cathelicidin	F930015N03, NM_004345, AF484553	+	+	+
				Hepcidin	2210420P15, NM_052971	+	+	-
				Histone 2A	9030420B16, NM_003512, 1190022L06, NM_021052	+	+	-
				Lysozyme	9530003J23, I420013M05, NM_012771	+	-	+
				MBP	2510004C07, HIX0009634.2, NM_031619	+	+	+
				Proenkaphalin	4922504O09, HIX0007519.2, NM_017139	+	+	+
				Secretogranin	5730420J08, HIX0015625.2 NM_012526	+	+	+
				VIP	9130007F05, HIX0006306.2	+	+	-
3	CCAGGG	Unknown	8	Alpha defensin	2010016B13, 2010016F14, NM_021010, NM_001925	+	+	-
				Spag11	9230111C08, NM_145087	+	-	+
				BPI	9230105K17, BC040955	+	+	-
				DBI	6720460E16, NM_020548	+	+	-
				Granulin	0610012H06, BC000324, NM_017113	+	+	+
				Lysozyme	9530003J23, I420013M05, AF099029, NM_012771	+	+	+
				Melanotropin alpha	5730403F20, NM_000939, NM_139326	+	+	+
				SPYY	0710005A05, C820007C10, HIX0006525.2, NM_012614	+	+	+
4	ACCTGG	Unknown	7	Betadefensin	9230107º10, AF525930, D630029A12, BC033298, NM_031810, NM_153324, 2310001F05, NM_004942, 1700011J22, NM_152250, 9230103N16, 4930563B01	+	+	+
				BPI	9230105K17, BC040955	+	+	-
				Calgranulin	F430201H11, NM_002965, NM_053587	+	+	+
				Cathelicidin	F930015N03, NM_004345,	+	+	+

					AF484553			
				Granulin	0610012H06, BC000324, NM_017113	+	+	+
				Lactoferrin	9830118D19, NM_002343	+	+	-
				ZAP	F420004O17, HIX0007129.3, NM_173045	+	+	+
5	ATGGAG	Nkx2-1	10	Alpha defensin	2010016B13, 2010016F14, NM_001926, NM_001925, NM_005217	+	+	-
				Calgranulin	F430201H11, NM_002965, NM_053587	+	+	+
				Cathelicidin	F930015N03, NM_004345, AF484553	+	+	+
				DBI	6720460E16, NM_020548	+	+	-
				Slpi	2310075E18, HIT000038907.2,NM_053372	+	+	+
				Hepcidin	2210420P15, NM_052971	+	+	-
				Lactoferrin	9830118D19, NM_002343	+	+	-
				MBP	2510004C07, HIX0009634.2, NM_031619	+	+	+
				VIP	9130007F05, HIX0006306.2	+	+	-
				Vasostatin	G630083O06, HIX0011909.2,NM_021655	+	+	+
6	TCTTTC	Unknown	9	Alpha defensin	2010016B13, 2010016F14, NM_001925, NM_005217	+	+	-
				BPI	9230105K17, BC040955	+	+	-
				Calgranulin	F430201H11, NM_002965, NM_053587	+	+	+
				Slpi	2310075E18, HIT000038907.2, NM_053372	+	+	+
				Hepcidin	2210420P15, NM_052971	+	+	-
				MBP	2510004C07, HIX0009634.2, NM_031619	+	+	+
				Melanotropin alpha	5730403F20, NM_000939, NM_139326	+	+	+

				SPYY	0710005A05, HIX0006525.2, NM_012614	+	+	+
				ZAP	F420004O17, HIX0007129.3 , NM_173045	+	+	+
7	TGGCATT	NF-1	9	Alpha defensin	2010016B13, 2010016F14 NM_021010, NM_001925 NM_005217	+	+	-
				Apoa2	I530003A11, NM_013112	+	-	+
				Beta defensin	AF525930, D630029A12, BC033298, NM_031810, 1700012K18, NM_153324, 2310001F05, NM_004942, 1700011J22, NM_152250, 9230103N16, 4930563B01	+	+	+
				Granulin	0610012H06, BC000324, NM_017113	+	+	+
				Lactoferrin	9830118D19, NM_002343	+	+	-
				Lysozyme	9530003J23, AF099029, NM_012771	+	+	+
				Melanotropin alpha	5730403F20, NM_000939, NM_139326	+	+	+
				SPYY	C820007C10, NM_012614	+	-	+
				Vasostatin	G630083O06, HIX0011909.2, NM_021655	+	+	+
8	CCCGCCCC	Sp1, Sp3	6	Alpha defensin	2010016B13, 2010016F14, NM_021010, NM_001926	+	+	-
				Apoa2	HIT000032344.2, NM_013112	-	+	+
				BPI	9230105K17, BC040955	+	+	-
				DBI	6720460E16, NM_020548	+	+	-
				Lactoferrin	9830118D19, NM_002343	+	+	-
				Melanotropin alpha	5730403F20, NM_000939, NM_139326	+	+	+
