# Supplementary material for: Computational promoter analysis of mouse, rat and human antimicrobial peptide-coding genes
Source: BMC Bioinformatics. 2006 Dec 18;7(Suppl 5):S8. doi: 10.1186/1471-2105-7-S5-S8 (PMC1764486; doi:10.1186/1471-2105-7-S5-S8)
Supplement: Additional file 5 — Supplementary tables 5A and 5B Distribution of motifs corresponding to different TF groups among AMP families and Ranking of TF groups according to their frequency of appearance in different AMP families. [file 1471-2105-7-S5-S8-S5.pdf]

Supplementary Table 5A: Distribution of motifs corresponding to different TF groups among AMP families. Tissue/function-specific TF groups are AD: adipocyte-related TFs; NHR: nuclear hormone receptor TFs; CC: cell cycle-related TFs; IMM: immune cell-specific TFs; LIV: liver cell-specific TFs; LUNG: lung cell-specific TFs; MUS: muscle cell-specific TFs; NS: nervous system-related TFs; PAN: pancreatic B-cell related; PIT: pituitary gland-specific TFs; Eye: eye-specific TFs; BS: bone-specific TFs. For each AMP family the TF group(s) with the highest number of motifs is (are) underlined.

AMP Families	AD	NHR	CC	IMM	LIV	LUNG	MUS	NS	PAN	PIT	EYE	BS
Alphadefensin	6	12	2	6	6	5	3	9	2	1	0	0
Apoa2	5	5	5	4	5	5	4	6	4	3	0	0
Betadefensin	6	5	4	6	6	5	2	8	2	3	0	1
bin1b/spag11	9	9	3	5	10	10	6	10	5	2	0	3
Bpi	6	7	8	8	8	5	8	7	7	4	0	0
Calgranulin	8	5	6	9	9	6	7	11	7	5	1	2
Cathelicidin	4	8	6	8	5	5	1	6	3	5	0	1
Dbi	7	8	4	5	7	6	4	6	1	4	0	0
Slpi	6	7	3	6	6	5	3	8	5	4	0	0
Granulin	6	5	6	4	6	6	4	5	4	3	0	0
Hepcidin	10	9	3	7	11	11	3	9	6	7	0	0
Histone	5	2	3	3	5	4	3	3	4	3	0	0
Lactoferrin	7	10	3	4	7	6	4	8	3	1	0	0
Lysozyme	4	2	4	3	4	4	3	3	2	1	0	0
Mbp	6	7	9	9	7	6	6	7	2	6	0	2
Melanotropinalpha	9	6	9	7	8	7	4	8	3	4	0	0
Proenkaphalin	7	7	3	4	7	7	1	8	3	3	0	1
Secretogranin	1	5	2	6	3	2	4	3	3	3	0	1
Spyy	5	5	2	5	5	3	1	5	3	5	0	0
Vip	3	3	3	4	3	2	1	3	3	1	0	1
Vstn	4	4	3	3	5	4	3	4	1	2	0	0
Zap	7	8	6	6	8	8	3	6	4	4	0	0

Supplementary Table 5B. Ranking of TF groups according to their frequency of appearance in different AMP families. For example, under rank 1, AD is the the most frequently occurring TF group in five of the AMP families that are listed in Table S2a. Underlined numbers indicate the high-ranking liver-specific, nervous system-related, adipocyte-related, nuclear hormone-related, immune cell-specific and lung-specific TFs.

Rank	Tissue/Function-specific TF groups											
	AD	NHR	CC	IMM	LIV	LUNG	MUS	NS	PAN	PIT	EYE	BS
1	5	6	5	6	9	5	1	7	0	1	0	0
2	6	6	2	2	6	3	0	4	1	0	0	0
3	4	1	1	2	5	1	1	3	1	0	0	0
4	3	2	0	0	1	1	0	3	1	1	0	0
5	0	3	2	4	1	4	3	5	2	2	0	0
6	1	0	1	5	0	3	4	0	1	2	0	0
7	0	1	4	3	0	2	4	0	5	2	0	0
8	2	1	3	0	0	2	1	0	5	4	0	0
9	0	1	4	0	0	1	4	0	2	3	0	2
10	1	1	0	0	0	0	4	0	4	6	0	4
11	0	0	0	0	0	0	0	0	0	1	14	16
12	0	0	0	0	0	0	0	0	0	0	8	0
Avg.	3.32	3.55	5.27	3.95	2.05	4.32	7.05	2.77	7.18	7.77	11.36	10.64
