# Supplementary material for: Computational promoter analysis of mouse, rat and human antimicrobial peptide-coding genes
Source: BMC Bioinformatics. 2006 Dec 18;7(Suppl 5):S8. doi: 10.1186/1471-2105-7-S5-S8 (PMC1764486; doi:10.1186/1471-2105-7-S5-S8)
Supplement: Additional file 6 — Supplementary table 6. Distribution of individual transcription factors among AMP families. [file 1471-2105-7-S5-S8-S6.pdf]

Supplementary Table 6. Distribution of individual transcription factors among AMP families

Transcription Factor (TF)	No. AMP families with detected TF	AMP family names
GR	20	alpha defensin, apoa2, betadefensin, bin1b, bpi, calgranulin, cathelicidin, dbi, slpi, granulin, hepcidin, histone, lactoferrin, lysozyme, mbp, melanotropinalpha, penk1, vip, vasostatin, zap
RXR-alpha	18	alpha defensin, betadefensin, bpi, calgranulin, cathelicidin, dbi, slpi, granulin, hepcidin, histone, lactoferrin, mbp, melanotropinalpha, penk1, secretogranin, spyy, vip, zap
AR	17	alpha defensin, apoa2, betadefensin, bin1b, bpi, calgranulin, cathelicidin, dbI, slpi, granulin, hepcidin, lactoferrin,<br>mbp, melanotropinalpha, penk1, vasostatin, zap
Sp1	16	apoa2, bpi, calgranulin, dbi, granulin, hepcidin, histone, lactoferrin, lysozyme, mbp, melanotropinalpha, penk1, secretogranin, spyy, vasostatin, zap
VDR	16	alpha defensin, apoa2, betadefensin, bpi, calgranulin, cathelicidin, dbi, slpi, granulin, hepcidin, lactoferrin, mbp, secretogranin, spyy, vasostatin, zap
T3R-alpha	15	alpha defensin, apoa2, betadefensin, bin1b, bpi, calgranulin, cathelicidin, dbi, slpi, hepcidin, mbp, melanotropinalpha, penk1, spyy, zap
Meis-1a	15	alpha defensin, apoa2, betadefensin, calgranulin, cathelicidin, dbi, slpi, granulin, histone, lactoferrin, lysozyme, mbp, secretogranin, spyy, vip
Meis-1b	15	alpha defensin, apoa2, betadefensin, bin1b, calgranulin, cathelicidin, dbi, slpi, granulin, histone, lysozyme, mbp, secretogranin, spyy, vip
RAR-alpha1	14	alpha defensin, apoa2, betadefensin, bpi, calgranulin, cathelicidin, dbi, slpi, hepcidin, mbp, penk1,

		secretogranin, spyy, zap
LXR-alpha:RXR-alpha	13	alpha defensin, apoa2, betadefensin, bpi, calgranulin, cathelicidin, dbi, slpi, lactoferrin, mbp, secretogranin, vip, zap
NF-1	13	apoa2, bin1b, calgranulin, cathelicidin, dbi, granulin, histone, lactoferrin, lysozyme, mbp, melanotropinalpha, vip, zap
AP-2alphaA	13	apoa2, bin1b, bpi, cathelicidin, slpi, granulin, hepcidin, lysozyme, mbp, melanotropinalpha, penk1, spyy, vasostatin
Nkx2-1	12	betadefensin, bin1b, bpi, calgranulin, cathelicidin, dbi, slpi, granulin, lysozyme, spyy, vip, zap
c-Myb	12	bpi, calgranulin, cathelicidin, dbi, slpi, granulin, lysozyme, mbp, melanotropinalpha, vip, vasostatin, zap

GR is involved in the regulation of numerous physiological processes including lymphocyte apoptosis, T cell development and inflammatory responses [38]. Several of the TFs found in our analysis are known to interact with GR, like AP-1, c-Ets-2 etc. AR has also been shown to play role in the immune response. It appears that androgens have an influence on the developmental maturation of T and B lymphocytes [39]. RXR-alpha binds to many other TFs forming complexes that can regulate multiple pathways, including immunomodulatory pathways. It has been shown that RXR-alpha binds to VDR, forming a heterodimer that inhibits NF-AT and plays a role in immunosuppression [40]. RXR-alpha also binds to PPAR-gamma and causes an apoptotic signaling cascade in B cells through NF-kappaB activation [41].

VDR is the receptor protein for 1,25-dihydroxyvitamin D which is involved in regulating cell growth, modulating the immune system and the renin-angiotensin system [42]. VDR which was identified in our analysis among beta-defensins was shown to mediate the induction antimicrobial peptide gene expression in human like beta-defensin 2 [43]. T3R-alpha, another frequently occurring TF, covers 15 of the AMPcg families. T3R-alpha binds to thyroid hormone and is involved in the control of B-cell production level [44]. RAR-alpha1, a receptor for retinoids is constitutively produced in adenoidal T and B cells [45]. LXR-alpha:RXR-alpha heterodimers function as sensors for cellular oxysterols and are transcriptional activators of genes that control sterol and fatty acid metabolism/homeostasis [46]. In summary, the occurrence of different members of NHR family as most frequently occurring TFs among AMPcg families indicates an intricate regulatory network encompassing the endocrine (i.e. lipid metabolism) system and innate immunity (see Supplementary Table 6).

NF-1 (nuclear factor 1) is known to be involved in regulation of genes associated with adipogenesis and signal transduction pathways induced by steroid hormones like vitamin D, thyrotropin [47]. The AMP member diazepam binding inhibitor (Dbi) carries a NF-1 site that plays a crucial role lipogenesis associated transcription [48].

References

38. Reichardt HM. **Immunomodulatory activities of glucocorticoids: insights from transgenesis and gene targeting**. *Curr Pharm Des* 2004, **10**: 2797-2805.
39. Olsen NJ, Kovacs WJ. **Effects of androgens on T and B lymphocyte development**. *Immunol Res* 2001, **23**: 281-288.
40. Takeuchi A, Reddy GS, Kobayashi T, Okano T, Park J, et al. **Nuclear factor of activated T cells (NFAT) as a molecular target for 1alpha,25-dihydroxyvitamin D3-mediated effects**. *J Immunol* 1998, **160**: 209-218.
41. Schlezinger JJ, Jensen BA, Mann KK, Ryu HY, Sherr DH. Peroxisome proliferator-activated receptor gamma-mediated NF-kappa B activation and apoptosis in pre-B cells. *J Immunol* 2002, **169**: 6831-6841.
42. Holick MF. **Evolution and function of vitamin D.Recent Results.** *Cancer Res* 2003, **164**: 3-28.
43. Wang TT, Nestel FP, Bourdeau V, Nagai Y, Wang Q, et al. **Cutting edge: 1,25-dihydroxyvitamin D3 is a direct inducer of antimicrobial peptide gene expression**. *J Immunol* 2004, **173**: 2909-2912,
44. Arpin C, Pihlgren M, Fraichard A, Aubert D, Samarut J, et al. **Effects of T3R alpha 1 and T3R alpha 2 gene deletion on T and B lymphocyte development**. *J Immunol* 2000, **164**: 152-160.
45. Ballow M, Wang X, Xiang S, Allen C. **Expression and regulation of nuclear retinoic acid receptors in human lymphoid cells**. *J Clin Immunol* 2003, **23**: 46-54.
46. Edwards PA, Kennedy MA,Mak PA. **LXRs; oxysterol-activated nuclear receptors that regulate genes controlling lipid homeostasis**.*Vascul Pharmacol* 2002, **38**: 249-256.
47. Gronostajski RM. **Roles of the NFI/CTF gene family in transcription and development**. *Gene* 2000, **249**: 31-45.
48. Hansen HO, Andreasen PH, Mandrup S, Kristiansen K, Knudsen J. I**nduction of acyl-CoA-binding protein and its mRNA in 3T3-L1 cells by insulin during preadipocyte-to-adipocyte differentiation**. *Biochem J* 1991, **277**: 341-344.
