# Supplementary material for: Computational promoter analysis of mouse, rat and human antimicrobial peptide-coding genes
Source: BMC Bioinformatics. 2006 Dec 18;7(Suppl 5):S8. doi: 10.1186/1471-2105-7-S5-S8 (PMC1764486; doi:10.1186/1471-2105-7-S5-S8)
Supplement: Additional file 7 — Supplementary table 7. Common motifs detected among groups of enteris and myeloid-specific alpha-defensins. [file 1471-2105-7-S5-S8-S7.pdf]

Supplementary Table 7. **Common motifs detected among groups of enteric and myeloid-specific alpha-defensins**. The species abbreviations are Rano: *Rattus norvegicus*, Mumu: *Mus musculus*; Patr: *Pan troglodytes*; Hosa: *Homo sapiens*. Unknown: motif does not match any of the TRANSFAC-listed TF binding sites.

Common motif	Consensus motif	Putative TF binding sites	Gene
20	AGAARCTCAGS	AR, PXR-1:RXR-alpha	*Hosa-DEFA4* (myeloid), *Patr-DEFA4* (myeloid), *Hosa-DEFA3* (myeloid), *Patr-DEFA5* (enteric)
10	CATAMTACCTGA	AP-1, c-Jun	*Hosa-DEFA4* (myeloid), *Patr-DEFA4* (myeloid), *Hosa-DEFA3* (myeloid), *Patr-DEFA5* (enteric)
11	KAGYTTTTWTCC	GATA-1, NF-AT1, NF-AT2, GATA-6, GATA-3, NF-AT3, NF-AT4	*Hosa-DEFA4* (myeloid), *Patr-DEFA4* (myeloid), *Hosa-DEFA3* (myeloid), *Patr-DEFA5* (enteric)
19	AGTAAAGCCA	Unknown	*Hosa-DEFA4* (myeloid), *Patr-DEFA4* (myeloid), *Hosa-DEFA3* (myeloid), *Patr-DEFA5* (enteric)
20	AGAARCTCAGS	YY1, STAT5A	*Rano-Def1* (myeloid), *Rano-Defcr4* (enteric)
17	GMASTTCTKT	IRF-1, IRF-3, NF-AT1, NF-AT2, NF-AT3, NF-AT4	*Rano-Def1* (myeloid), *Rano-Defcr4* (enteric)
6	GAAAAAAGAAT	Unknown	*Rano-Def1* (myeloid), *Rano-Defcr4* (enteric)
20	AGAARCTCAGS	AR, PXR-1:RXR-alpha	*Rano-Def1* (myeloid), *Mumu-Defcr2* (enteric)
7	AAAMATYCAT	POU1F1a, POU2F1	*Rano-Def1* (myeloid), *Mumu-Defcr2* (enteric)
4	GAAGGACCAGC	RAR-alpha1, RXR-alpha	*Rano-Def1* (myeloid), *Mumu-Defcr2* (enteric)
17	GMASTTCTKT	GR, AR	*Mumu-Defcr20* (enteric), *Mumu-Defcr2* (enteric)
3	ATTCTCHTGGACA	RXR-beta, T3R-alpha1, T3R-beta1, USF1b, USF1 GR	*Mumu-Defcr20* (enteric), *Mumu-Defcr2* (enteric)
9	CTCTTGCCTG	C/EBPalpha	*Mumu-Defcr20* (enteric), *Mumu-Defcr2* (enteric)
13	GGAATCAAGT	Unknown	*Mumu-Defcr20* (enteric), *Mumu-Defcr2* (enteric)
10	CATAMTACCTGA	AP-1, c-Jun	*Mumu-Defcr20* (enteric), *Mumu-Defcr2* (enteric)
5	CCTGCTCCCTGBT	AR, T3R-alpha, RXR-alpha, VDR	Mumu-Defcr20 (enteric), Mumu-Defcr2 (enteric)
8	TGTCCTGGTCC	GR, PR-alpha, PR-beta, PR B, RAR-alpha1, RXR-beta RAR-gamma, T3R-alpha, T3R-beta1, T3R-beta2, HNF-4alpha, RAR-alpha, RAR-alpha:RXR-gamma, RAR-beta, RAR-beta:RXR-alpha, AR, NFI/CTF, RXR-alpha, VDR, ERR1	*Mumu-Defcr20* (enteric), *Mumu-Defcr2* (enteric)
4	GAAGGACCAGC	RAR-alpha1, RXR-alpha	*Mumu-Defcr20* (enteric), *Mumu-Defcr2* (enteric)
