# Supplementary material for: Computational promoter analysis of mouse, rat and human antimicrobial peptide-coding genes
Source: BMC Bioinformatics. 2006 Dec 18;7(Suppl 5):S8. doi: 10.1186/1471-2105-7-S5-S8 (PMC1764486; doi:10.1186/1471-2105-7-S5-S8)
Supplement: Additional file 8 — Supplementary table 8. Motif distribution across the rat Defcr4, Defa6, Defa8 and Defa9 promoter regions. [file 1471-2105-7-S5-S8-S8.pdf]

Supplementary Table 8: Motif distribution across the rat *Defcr4, Defa6, Defa8* and *Defa9* promoter regions. The first four columns show the motif numbers that are present in the *Defcr4*, *Defa6*, *Defa8* and *Defa9* promoters. The last column shows the corresponding TF binding sites for each of the motifs. Not Found: the motif is absent from the promoter region of the AMPcg. Unknown: motif does not match any of the TRANSFAC-listed TF binding sites.

Defcr4 motif no.	Defa6 motif no.	Defa8 motif no.	Defa9 motif no.	TF binding sites
1	1	1	1	COUP-TF2, ERR1, AR, GR, NF-E3, FXR, NP-IV
2	2	2	2	Nkx2-1
3	3	3	3	STAT5A, STAT6
Not found	4	4	4	RXR-alpha, RAR-alpha1, GR, HNF-4alpha, RAR-beta
Not found	5	5	5	POU2F1, CDX2, HNF-3, Crx, FOXJ2
Not found	6	6	6	c-Ets-1, c-Ets-2, CLIM2, c-Myc, COUP-TF1, COUP-TF2, CP2, E12, E47, EMF1, ERR1, FXR:RXR-alpha, Hp55, MITF, M-Twist, NeuroD, PPAR-gamma, RAR-alpha1, Tal-1, TLX, USF1, USF-1, USF1:USF2, VDR
7	7	7	7	HOXA9,Xvent-2,Meis-1a
Not Found	8	8	8	Unknown
9	9	9	9	Irx4, MAZ, PPAR-alpha, R1, R2, RXRalpha, Sp1, VDR
Not found	10	10	10	CXR, ER-alpha, GR, RAR-alpha, RAR-alpha1, RXR-alpha, RXR-gamma, Sox13, Sox17, TR2-11, VDR
11	11	11	11	Nkx2-1,Meis-1a,Nrf2
Not found	12	12	12	COUP-TF2, ERR1, AR, COUP-TF2, COUP-TF2, Nkx2-8, Nkx2-1, Nkx2-8, Nkx2-1, FXR, NF-E3, NF-E3

13	13	13	13	c-Fos, Nkx2-1, c-Jun, GR
14	14	14	14	Unknown
Not found	15	15	15	HNF-4alpha
16	16	16	16	BXR-beta, CAR:RXR-alpha, CAR2:RXR-alpha, COUP-TF1, COUP-TF2, ER-alpha, ERR1, ERRalpha1, FXR, FXR:RXR-alpha, GCNF, GR-alpha, LXR-alpha, LXR-beta, Nur77, PPAR-alpha, PXR-1:RXR-alpha, RAR-alpha1, RAR-beta, REVERB-alpha, RORalpha1, RXR-alpha, RXR-beta, SF-1, T3R-alpha, T3R-alpha1, T3R-beta1, TGACCT, TR2-11
17	17	17	17	GR
18	18	18	18	LVa, GR, POU1F1a, GR, AR
19	19	19	19	Sp1,VDR, AP-2alphaA, LEF-1
Not found	20	20	20	HNF-4, TR2-11, T3R-alpha, COUP-TF1
