# Supplementary material for: Computational promoter analysis of mouse, rat and human antimicrobial peptide-coding genes
Source: BMC Bioinformatics. 2006 Dec 18;7(Suppl 5):S8. doi: 10.1186/1471-2105-7-S5-S8 (PMC1764486; doi:10.1186/1471-2105-7-S5-S8)
Supplement: Additional file 9 — Supplementary tables 9A and 9B. TF binding sites that correspond to ab initio-predicted motifs derived from the Penk family promoter regions and Motif arrangements in promoter region in mouse (4922504O09), human (HIX0007519.2) and rat (NM_017139) of Penk family members. [file 1471-2105-7-S5-S8-S9.pdf]

Supplementary Table 9A. TF binding sites that correspond to *ab initio*-predicted motifs derived from the Penk family promoter regions.

Motif no.	Motif Occurrence	Motif	TF binding sites
1	3	CCAGTAACCTGCG	FXR:RXR-alpha, LXR-alpha:RXR-alpha, LXR-beta:RXR-alpha, ERRalpha1
2	3	TATAAAGTGGCTGT	TFIID, TBP
3	3	GATCTAAAGAAGAAA	AR, GR
4	3	CCAAGTCCGTC	SF-1, GR
5	3	TTAAGATCCCCA	NF-kappaB1, NF-kappaB2, NF-kappaB2 precursor, AP-2alpha, AP-2alphaA
6	3	GTGATDCAGGA	AP-1, c-Fos, c-Jun, JunD
7	3	TCCAGVAAGDH	c-Ets-1, Elk-1, SAP-1a, SAP-1b, SRF, PEA3, ELF-1
8	3	CAGGCGTCGGCGCG	DREB1A, ZF5, E2F
9	3	CGATTGGGGCGCGC	NFI/CTF, CTF, NF-Y
10	3	CCAGAVAGGCAG	UBP-1, GATA-1, GATA-3, Meis-1a, Meis-1b, GATA-4, RXR-beta, VDR, MOT3
11	3	CCGGGTCCTA	Unknown
12	3	AGCCCGTGBC	USF-1, USF1, USF2, USF2b, USF, HMBP, EmBP-1a
13	3	GTGACTTTGCCCCA	DSF, GCN4, COUP-TF1, RAR-beta, RXR-alpha, RAR-alpha1, TLX, Pax-2.1, Pax-2.2, IRF-4, IRF-8, AP-2alpha, AP-2alphaA, C/EBPgamma, PPAR-gamma:RXR-alpha, VDR, LXR-alpha:RXR-alpha
14	3	GATCTGTBTT	Sox2, Meis-1a, Meis-1b, GR
15	3	TGAAATTTGG	Unknown
16	3	GCTGTGGGGACGTCC	AML1, AML1a, AML1c, MZF1, MIG1, MZF-1, AP-2alpha, AP-2alphaA, MBP-1, NF-kappaB1, NF-kappaB2, NF-kappaB2 precursor
17	3	BHHCAAGAGGA	Unknown
18	3	GGAAGGGGCAG	VDR, LXR-alpha:RXR-alpha, CAC-binding protein, NF-E2, PPAR-gamma:RXR-alpha, Sp1
19	3	AHGCCCCAACC	Sp1, PPAR-gamma:RXR-alpha, VDR, LXR-alpha:RXR-alpha, AP-2alphaA, ADR1 C/EBPalpha, C/EBPbeta
20	3	GGACAGGATG	Meis-1a, Meis-1b, Elk-1, SAP-1a, SAP-1b, SRF, E47, Fli-1, Net, TCF

All motifs were detected in mouse, rat and human sequences. The underlined TF binding sites are known to bind TFs in the proenkephalin promoter region [24][25][26][27]. The species abbreviations are Hs: *Homo sapiens*; Mm: *Mus musculus*: Rn: *Rattus norvegicus*. Unknown: motif does not match any of the TRANSFAC-listed TF binding sites.

24. Liu F, Kondova I, Kilpatrick DL. **Detection of PACH1, a nuclear factor implicated in the transcriptional regulation of meiotic and early haploid stages of spermatogenesis**. *Mol Reprod Dev* 2000, **57**: 224-231.
25. Kobierski LA, Wong AE, Srivastava S, Borsook D, Hyman SE. **Cyclic AMP-dependent activation of the proenkephalin gene requires phosphorylation of CREB at serine-133 and a Src-related kinase**. *J Neurochem* 1999, **73**: 129-138.
26. Fu W, Shah SR, Jiang H, Hilt DC, Dave HP, et al. **Transactivation of proenkephalin gene by HTLV-1 tax1 protein in glial cells: involvement of Fos/Jun complex at an AP-1 element in the proenkephalin gene promoter**. *J Neurovirol* 1997, **3**: 16-27.
27. Le Y, Gagneten S, Larson T, Santha E, Dobi A, et al. **Far-upstream elements are dispensable for tissue-specific proenkephalin expression using a Cre-mediated knock-in strategy**. *J Neurochem* 2003, **84**: 689-697.

Supplementary Table 9B. Motif arrangements in promoter region in mouse (4922504O09), human (HIX0007519.2) and rat (NM_017139) of Penk family members. The species abbreviations are Hs: *Homo sapiens*; Mm: *Mus musculus*: Rn: *Rattus norvegicus*.

Species	Motif arrangement
Hs,Rn	3-5-15-18-4-16-8-9-2-10-1-13
Mm, Hs	7-12-3-5-1-13
Hs, Rn	3-5-1-13-20
Mm, Hs, Rn	3-5-1-13
