# Supplementary material for: Computational promoter analysis of mouse, rat and human antimicrobial peptide-coding genes
Source: BMC Bioinformatics. 2006 Dec 18;7(Suppl 5):S8. doi: 10.1186/1471-2105-7-S5-S8 (PMC1764486; doi:10.1186/1471-2105-7-S5-S8)
Supplement: Additional file 10 — Supplementary tables 10A and 10B. TF binding sites that correspond to ab initio-predicted motifs derived frokm Zap family promoter regions and Promoter motif arrangements in mouse (FA20004O17), human (HIX0007129.3) and rat (NM_173045) Zap family members. [file 1471-2105-7-S5-S8-S10.pdf]

Supplementary Table 10A. TF binding sites that correspond to *ab initio*-predicted motifs derived from Zap family promoter regions. The species abbreviations are Hs: *Homo sapiens*; Mm: *Mus musculus*, Rn: *Rattus norvegicus*. Unknown: motif does not match any of the TRANSFAC-listed TF binding sites.

Motif No.	Motif Occurrence	Species	Motif	TF binding sites
1	3	Mm, Hs, Rn	CTCCACCTGTTCCTT	Alfin1, RXR-alpha, VDR, E12, E47, MyoD, myogenin, EMF1, EMF2, EMF3, EMF4, Myf-5, c-Myc, USF2, CAN, E2A, DEP2, HEB, Ac, AS-C, T3, Da, Sc, Sn, CLIM2, GATA-1, Lmo2, Tal-1, USF-1, NeuroD, NEUROD, LVa, PR B, AR, GR, c-Ets-2, ESE-1, HELIOS, LyF-1
2	3	Mm, Hs, Rn	TCACCGCACT	ER-alpha, ABI4, AML1a
3	3	Mm, Hs, Rn	CTGGGGGGCCC	MIG1, Sp1, ZAC-1a
4	3	Mm, Hs, Rn	AAGCAGTTGGT	c-Myb, c-Myc, E47, NeuroD, NEUROD, E12, MyoD, MyoD:E12, myogenin, Myogenin:E12, DEC2, c-Myb:HES-1
5	3	Mm, Hs, Rn	GGCTCTTTAATT	AR, GR, LF-A1, RAR-alpha1, RAR-beta, RAR-gamma, RORalpha1, RXR-beta2, LXR-alpha:RXR-alpha, LXR-beta:RXR-alpha, T3R-alpha, FXR:RXR-alpha, PXR-1:RXR-alpha, COUP, FOR1, FOR2, ER-alpha, AP-1, RXR-alpha, TAF(II)28, LXR-alpha, VDR, TR2-11, PPAR-gamma
6	3	Mm, Hs, Rn	CATGACCCTGGAG	RXR-gamma, CAR:RXR-alpha, Nkx2-1
7	3	Mm, Hs, Rn	ACTCTAAGGTAT	Unknown
8	3	Mm, Hs, Rn	ATTCGCTCTCCC	LyF-1, RXR-beta,VDR
9	3	Mm, Hs, Rn	GGTTTACCTT	CAR:RXR-alpha, LXR-alpha:RXR-alpha, SXR, RAR-beta, RAR-gamma, RXR-alpha, RAR-alpha1, ER-alpha
10	3	Mm, Hs, Rn	GAGCGGCACC	Unknown
11	3	Mm, Hs, Rn	AATATCCAAG	NF-1, TGGCA-binding protein

12	3	Mm, Hs, Rn	AGCAGCATCA	Unknown
13	3	Mm, Hs, Rn	GAGAGTAACAA	GATA-6, GCN4, PR B
14	3	Mm, Hs, Rn	AATAGGACTT	GR
15	3	Mm, Hs, Rn	CGGATTTGAGGACGC	Unknown
16	3	Mm, Hs, Rn	AAAATCATCTT	Otx2, GATA-3
17	3	Mm, Rn	TAAGTTTCGATTCT	Unknown
18	3	Mm, Hs, Rn	GGAGTCTGGAGG	Nkx2-1
19	3	Mm, Rn	GAGTTGGAAAGCGA	NF-AT1, NF-1, Ftz
20	3	Mm, Hs, Rn	GTGCGCCCACGG	MTF-1

Supplementary Table 10B. Promoter motif arrangements in mouse (F420004O17), human (HIX0007129.3) and rat (NM_173045) Zap family members. The species abbreviations are Hs: *Homo sapiens*; Mm: *Mus musculus*: Rn: *Rattus norvegicus*.

**Species**	**Motif arrangement**
Mm, Hs	5-7-9-16-4-13-2-1-11-15-8-19-10-17-20-6
Mm, Hs	1-11-15-8-10-20
Hs, Rn	1-11-15-8-10-20-18
Mm, Hs, Rn	1-11-15-8-10-20
