# Supplementary material for: Computational promoter analysis of mouse, rat and human antimicrobial peptide-coding genes
Source: BMC Bioinformatics. 2006 Dec 18;7(Suppl 5):S8. doi: 10.1186/1471-2105-7-S5-S8 (PMC1764486; doi:10.1186/1471-2105-7-S5-S8)
Supplement: Additional file 11 — Supplementary table 11. P-value table of motif groups. [file 1471-2105-7-S5-S8-S11.pdf]

Supplementary Table 11. P-value table of motif groups. The row with bold-face values indicates the boundary of eleven AMP families that were significantly enriched in predicted NHR-binding motifs relative to the whole AMP family set.

No. of NHR-binding motif candidates in subpopulation	No. of motifs from all families included in subpopulations	No. of NHR-binding motif candiates	Total population (motifs from all families)	Bonferroni correction factor	p-value	Bonferroni corrected p-value	No. of groups included in statistically significant set
137	420	139	440	440	0.023154572	1	21
135	400	139	440	440	0.000884858	0.389337708	20
132	380	139	440	440	0.000128334	0.056466838	19
128	360	139	440	440	5.62042E-05	0.024729858	18
123	340	139	440	440	5.99005E-05	0.026356217	17
118	320	139	440	440	4.74915E-05	0.020896267	16
113	300	139	440	440	3.08239E-05	0.013562527	15
108	280	139	440	440	1.69494E-05	0.00745775	14
103	260	139	440	440	7.94636E-06	0.0034964	13
98	240	139	440	440	3.14129E-06	0.001382167	12
**92**	**220**	**139**	**440**	**440**	**2.81167E-06**	**0.001237134**	**11**
85	200	139	440	440	5.55134E-06	0.002442591	10
78	180	139	440	440	9.01694E-06	0.003967454	9
71	160	139	440	440	1.22648E-05	0.005396502	8
64	140	139	440	440	1.39428E-05	0.006134828	7
56	120	139	440	440	3.3908E-05	0.014919519	6
48	100	139	440	440	6.8438E-05	0.030112705	5
40	80	139	440	440	0.000112308	0.049415705	4
31	60	139	440	440	0.000399135	0.175619353	3

22	40	139	440	440	0.001106333	0.486786677	2
12	20	139	440	440	0.006893247	1	1
