# Supplementary material for: Computational promoter analysis of mouse, rat and human antimicrobial peptide-coding genes
Source: BMC Bioinformatics. 2006 Dec 18;7(Suppl 5):S8. doi: 10.1186/1471-2105-7-S5-S8 (PMC1764486; doi:10.1186/1471-2105-7-S5-S8)
Supplement: Additional file 1 — Supplementary table 1. FANTOM3 dataset-derived AMP transcripts which were new to mouse and absent in human. [file 1471-2105-7-S5-S8-S1.pdf]

Supplementary Table 1. FANTOM3 dataset-derived AMP transcripts which were new to mouse and absent in human.

**Riken clone ID/ GenBank acession**	**Gene Symbol**
D730003B11	*Csnd*
D730017I01	*Csnd*
D730018F19	*Csnd*
D730018I02	*Csnd*
D730032K03	*Csnd*
D730045O16	*Csnd*
D730048M03	*Csnd*
2010300L12	*Defcr-rs1*
2010319H24	*Defcr-rs1*
5033416M10	*Mcpt2*
G630050E22	*Mcpt4*
9030622B11	*Mcpt8*
0610031H01	*Hist2h2aa2*
1700048I17	*Hist2h2aa2*
